# PTPN1 downregulation contributes to pachymic acid-induced mitochondrial apoptosis in colorectal cancer cells

**DOI:** 10.3389/fonc.2026.1891182

**Published:** 2026-10-13

**Authors:** Chuang Liu, Qiang Su, Zhenxiang An, Sufang Zhou, Wen Che

**Affiliations:** 1 The First Clinical Medical College, Guizhou University of Traditional Chinese Medicine, Guiyang, China; 2 Department of Gastroenterology, First Affiliated Hospital of Guizhou University of Traditional Chinese Medicine, Guiyang, China; 3 Department of Pathology, Ruijin Hospital, Shanghai Jiaotong University School of Medicine, Shanghai, China

**Keywords:** cell proliferation, colorectal cancer, mitochondria, pachymic acid, PTPN1

## Abstract

Colorectal cancer (CRC) remains a major cause of cancer-related mortality. Pachymic acid (PA), a bioactive triterpenoid derived from Poria cocos, exhibits antitumor activity, but its effects on CRC and the involvement of PTPN1 remain unclear. This study investigated whether PTPN1 downregulation contributes to PA-induced mitochondrial apoptosis in HCT-116 CRC cells. Cell proliferation and migration were evaluated using viability, DNA synthesis, colony formation, wound healing, and transwell assays. Intracellular reactive oxygen species (ROS), mitochondrial membrane potential, apoptosis-related proteins, and the subcellular distribution of cytochrome c and Bax were examined. PTPN1 expression and overexpression rescue experiments were used to assess its functional involvement. PA inhibited cell proliferation and migration, increased ROS levels, and reduced mitochondrial membrane potential. These changes were accompanied by increased Bax, Bim, and cleaved caspase-3 expression, decreased Bcl-2 expression, and cytochrome c release from mitochondria into the cytoplasm. PA also downregulated PTPN1 expression. PTPN1 overexpression attenuated the effects of PA on proliferation, migration, ROS accumulation, mitochondrial membrane potential, and apoptosis-related protein expression. It also attenuated cytochrome c redistribution. These findings support a contribution of PTPN1 downregulation to PA-induced mitochondrial apoptosis in HCT-116 cells. Validation in additional CRC models, normal colonic epithelial cells, and *in vivo* systems is needed to establish the generalizability and therapeutic relevance of these findings.

## Introduction

Colorectal cancer (CRC) is the third most commonly diagnosed malignancy and the second leading cause of cancer-related death worldwide (1, 2). Despite advancements in conventional therapies, the five-year survival rate for CRC remains below 20%, primarily due to its propensity for recurrence and metastasis (3, 4). In 2020, the World Health Organization reported approximately 1.93 million new CRC cases globally (5, 6). Despite ongoing progress in surgical techniques and targeted therapies, CRC prognosis remains unfavorable (7). Consequently, there is a pressing need for deeper insights into CRC pathogenesis and the underlying molecular mechanisms driving tumor metastasis.

Jianpijiedu Decoction (JPJD), a clinically established traditional Chinese medicine formula, has demonstrated in CRC research to prolong survival and enhance survival rates among patients with stage II and III CRC over time (8). This decoction primarily acts by inhibiting the xCT/GSH/GPX4 axis to induce ferroptosis, effectively reversing 5-FU resistance in CRC (9). One of the components in JPJD, Poria cocos, contains pachymic acid (PA) as its key active ingredient (10, 11). PA has been shown to upregulate TP53INP2 and promote TRAF6-induced ubiquitination of caspase-8, leading to the activation of the apoptotic pathway and inhibiting the development of renal cell carcinoma (RCC) (10). Additionally, PA significantly inhibits the viability of gastric cancer SGC-7901 cells by arresting the cell cycle in the G0/G1 phase and inducing apoptosis (12). In cervical cancer, PA exerts antitumor activity by inducing endoplasmic reticulum (ER) stress, mitochondrial dysfunction, and activating the AMPK pathway (13). However, the specific role of PA in CRC remains unexplored to date. Recent work has further shown that PA can suppress PI3K/Akt-dependent tumor progression in gastric cancer through both ferroptosis and network pharmacology-guided mechanisms (14, 15), and that in triple-negative breast cancer (TNBC), PA induces ferroptosis by regulating the PTGS2/NOS2/ABCB1 signaling axis, thereby significantly suppressing the proliferation, migration, and invasion capabilities of cancer cells (16), lending further support to PTPN1/PTP1B as a plausible and recurring molecular target of PA across tumor types.

Based on preliminary target prediction using SwissTargetPrediction and TargetNet, combined with a literature review of PA’s reported molecular targets, PTPN1 emerged as a candidate of interest and was selected for further investigation. Molecular docking techniques were employed to predict the potential binding mode of PA to PTPN1. Notably, PTP1B (PTPN1) expression is markedly elevated in CRC tissues compared to adjacent non-tumor tissues, and its levels correlate with tumor differentiation, invasion, lymph node metastasis, and TNM stage. Higher PTP1B expression is associated with poorer survival rates in CRC patients (17, 18). Additionally, Jamunone M (JM) has been shown to regulate mitochondria-mediated apoptosis through the PI3K/Akt pathway via PTP1B, leading to G0/G1 phase arrest in breast cancer (BC) cells (19, 20). Based on these findings, we hypothesized that PA might induce mitochondria-mediated apoptosis in CRC cells by downregulating PTPN1, thereby inhibiting tumor progression.

## Materials and methods

### Cell culture and transfection

The CRC cell line HCT-116 (catalog no. SNL-077) was obtained from Wuhan SUNNCELL Biotechnology Co., Ltd. (Wuhan, China) and cultured in RPMI 1640 medium containing 10% fetal bovine serum (FBS). All cells were incubated at 37 °C in a humidified 5% CO_2_ atmosphere. Next, overexpressed (oe)-PTPN1 was transiently transfected into HCT-116 cells using Lipofectamine 3000 (Invitrogen Inc., Carlsbad, CA, USA).

### CCK-8 cell viability assay

Cells undergoing exponential growth were seeded in 96-well plates at a density of 4,000 cells per well, with each well containing 100 μL of culture medium. Subsequently, at specified intervals after seeding, 10 μL of CCK-8 reagent was added to each well, followed by a 2-hour incubation period. The absorbance at a wavelength of 490 nm was then quantified using a microplate spectrophotometer.

### EdU assay

A total of 1.6 × 10^5 cells from each experimental group were plated onto glass coverslips and maintained in culture for a duration of 24 hours. For the purpose of labeling and visualizing newly synthesized DNA, the Click-iT EdU Imaging Kits sourced from Invitrogen (USA) were utilized, adhering strictly to the manufacturer’s instructions. The stained cells were then examined and imaged using an inverted fluorescence microscope manufactured by Olympus (Tokyo, Japan).

### Colony formation assay

Cells were seeded into 6-well plates at a density of 1.6 × 10^3 cells per well and incubated for a period of two weeks. Following this, the cells were fixed using 4% paraformaldehyde and subsequently stained with crystal violet. The colonies formed were enumerated to assess the cells’ survival rate *in vitro*.

### Wound healing

Cells were seeded in six-well plates and treated with the indicated reagents. When cells reached confluence, a uniform scratch was made using a 200 μL pipette tip. The plates were washed once with fresh medium to remove detached cells and incubated for 24 h, after which images were acquired to assess wound closure.

### Transwell migration

A Transwell migration assay (without Matrigel coating) was conducted utilizing 24-well cell culture inserts featuring a translucent PET membrane with a pore diameter of 8.0 micrometers, sourced from BD Biosciences (USA). Specifically, 1.8 × 10^5 cells suspended in 200 microliters of serum-free Dulbecco’s Modified Eagle Medium (DMEM) were introduced into the upper compartment, while the lower compartment was filled with 800 microliters of DMEM enriched with 10% fetal bovine serum (FBS). After a 24-hour incubation period, the cells that had migrated to the underside of the membrane were immobilized using 4% paraformaldehyde (PFA) for 20 minutes and subsequently stained with 0.1% crystal violet for detailed examination.

### ROS measurement

Intracellular reactive oxygen species (ROS) levels were detected using the fluorescent probe 2’,7’-dichlorodihydrofluorescein diacetate (DCFH-DA). Upon entering cells, DCFH-DA is deacetylated by nonspecific esterases and subsequently oxidized by ROS to produce the fluorescent product DCF. Approximately 6 × 10^5^ cells were incubated with 10 μM DCFH-DA in serum-free medium at 37 °C for 30 min, washed three times with PBS, and imaged. Fluorescence intensity was quantified using ImageJ software.

### Observation of mitochondrial membrane potential by Rhodamine 123

Mitochondrial membrane potential was assessed using Rhodamine 123 (Rh123), a fluorescent dye that accumulates in energized mitochondria and is lost upon membrane depolarization. Cells were incubated with 10 μg/mL Rh123 at 37 °C for 30 min, washed once with PBS, and examined by fluorescence microscopy using an excitation wavelength of 505 nm and an emission filter at 534 nm. Reduced Rh123 fluorescence indicates loss of mitochondrial membrane potential and is associated with the initiation of mitochondria-mediated apoptosis.

### Protein data bank database

The PDB serves as a repository of information pertaining to the spatial configurations of proteins (https://www.rcsb.org/).

### Molecular docking

The spatial architecture of the PTPN1 protein, identified by PDB ID: 1NWL, was retrieved from the Protein Data Bank (PDB) database accessible at https://www.rcsb.org/. Subsequently, molecular docking simulations were executed utilizing the AutoDock software to determine the binding affinity score of the optimal binding conformation.

### Real-time quantitative PCR

Isolation of total RNA from CRC cells was carried out using TRIzol reagent under conditions devoid of RNase contamination. RNA concentration and purity (OD260/OD280) were assessed using a NanoDrop-type spectrophotometer. cDNA was synthesized via a two-step protocol using HiScript II Q RT SuperMix for qPCR (+gDNA wiper) (catalog no. R223-01, Vazyme, China), which included a genomic DNA elimination step (42 °C, 2 min) prior to reverse transcription (50 °C 15 min, 85 °C 5 s). Real-time quantitative PCR was performed using ChamQ SYBR qPCR Master Mix (catalog no. Q311-02, Vazyme, China) on a QuantStudio 6 Real-Time PCR System (Applied Biosystems, USA). The reaction program consisted of an initial denaturation at 95 °C for 10 min, followed by 40 cycles of 95 °C for 15 s and 60 °C for 60 s, with a melt-curve stage appended to confirm amplification specificity. Three independent biological replicates (n = 3) were analyzed by qPCR. Primers (**Supplementary Table S1**) were synthesized by TSINGKE Biotech (China). Relative mRNA expression was calculated using the 2^-ΔΔCt method with β-actin as the internal reference gene.

### Western blot analysis

Total protein was extracted using RIPA lysis buffer (containing PMSF; catalog no. MA0151, Meilunbio, China) supplemented with protease and phosphatase inhibitors (catalog no. MB12707, Meilunbio, China). Protein concentration was determined using a BCA protein assay kit (Reagent A catalog no. G3422, Reagent B catalog no. G3522; GBCBIO, China). Equal amounts of protein (30 μg per lane) were separated by SDS-PAGE using 4–20% gradient precast gels and transferred onto PVDF membranes (0.45 μm, catalog no. IPVH00010, Millipore, USA). Membranes were blocked with 5% non-fat milk in TBST for 1 h at room temperature and incubated overnight at 4 °C with the primary antibodies listed in **Supplementary Table S2**, followed by incubation with HRP-conjugated secondary antibodies (see **Supplementary Table S2**) for 1 h at room temperature. Signals were visualized using an enhanced chemiluminescence (ECL) substrate (catalog no. PMK003, Pumei, China) and quantified using ImageJ software, with β-actin (for whole-cell and cytoplasmic lysates) or COX IV (for mitochondrial fractions) as loading controls.

### Statistical analysis

The data presented herein are expressed as the mean ± standard deviation (SD). Statistical evaluations were conducted utilizing the GraphPad Prism 7 software package. For comparisons across multiple groups, one-way ANOVA followed by Tukey’s multiple comparisons test was employed. Differences were deemed statistically significant when the p-value was less than 0.05. All experiments were performed in at least three independent biological replicates (n ≥ 3) unless otherwise specified.

## Results

### PA treatment inhibits CRC cell proliferation

We investigated the impact of PA exposure on the proliferative capacity of CRC cells. **Figure 1A** illustrates the chemical structure of PA. Subsequently, cell proliferation was assessed using CCK-8 and EdU staining assays, while colony formation was evaluated through colony formation assays. The findings revealed that with the increase in PA treatment concentration, the inhibitory effect on CRC cell proliferation and cell colony formation was more obvious (**Figures 1B–D**).

**Figure 1:**
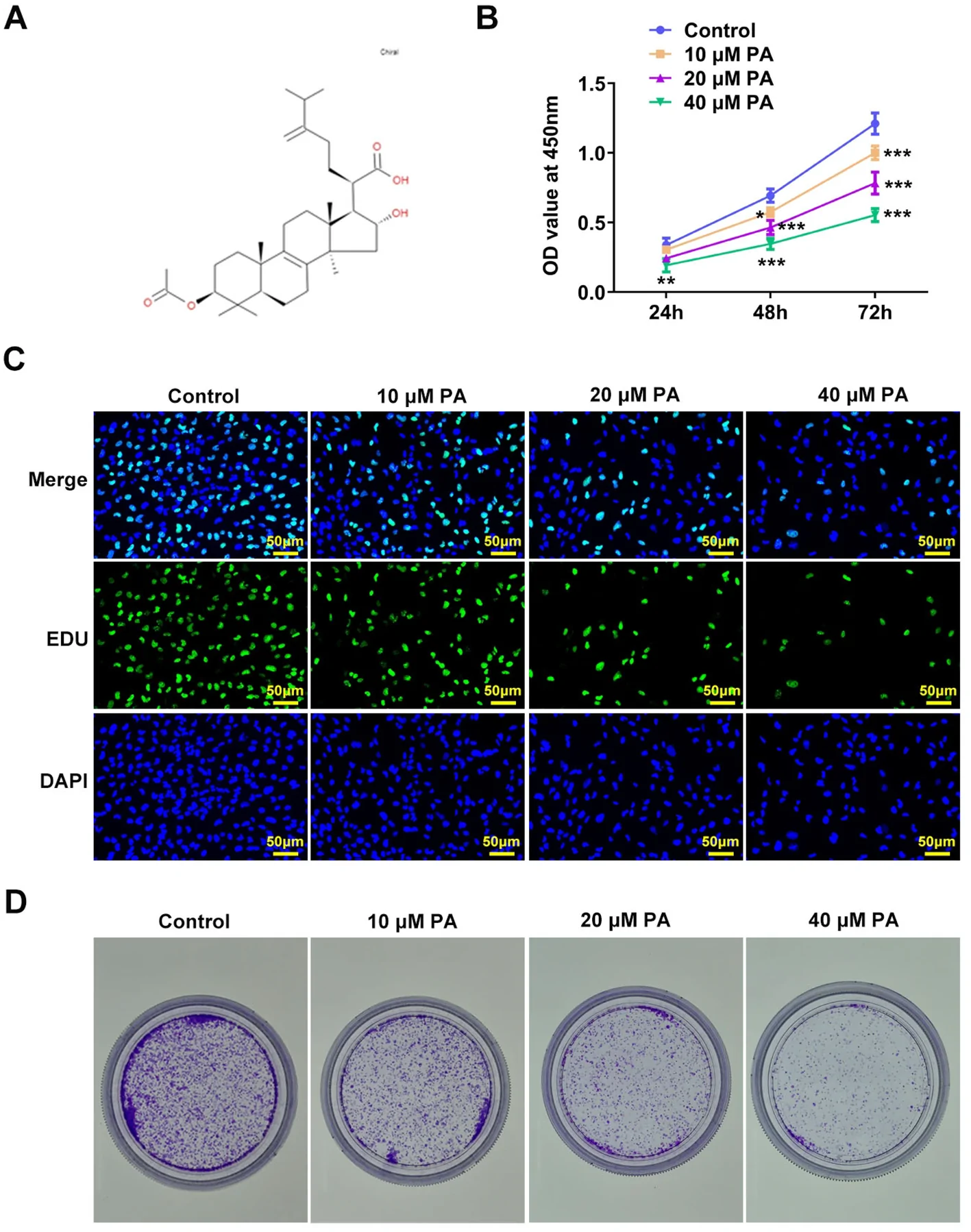
PA treatment inhibits the proliferation of CRC cells. **(A)** Chemical structure of PA. **(B)** CCK-8 assay showing dose-dependent reduction in cell viability at 24, 48, and 72 h. **(C)** EdU staining showing decreased DNA synthesis with increasing PA concentrations. **(D)** Colony formation assay showing reduced colony numbers after PA treatment. Data are presented as mean ± SD. *p < 0.05, **p < 0.01, ***p < 0.001 vs control.

### PA treatment inhibits the migration of CRC cells

To investigate the influence of PA treatment on the migratory behavior of CRC cells, we employed wound healing and transwell assays to assess cell migration under two complementary readouts. Additionally, western blot analysis was conducted to examine the expression levels of proteins associated with migration. Our findings indicated that PA treatment inhibited the expression of MMP2 and MMP9 in CRC cells, as well as cell migration in a dose-dependent manner (**Figures 2A, B**). Because these assays were conducted over a 24-h period comparable to the earliest proliferation readout, a partial contribution from reduced cell number cannot be fully excluded; this is discussed as a limitation below.

**Figure 2:**
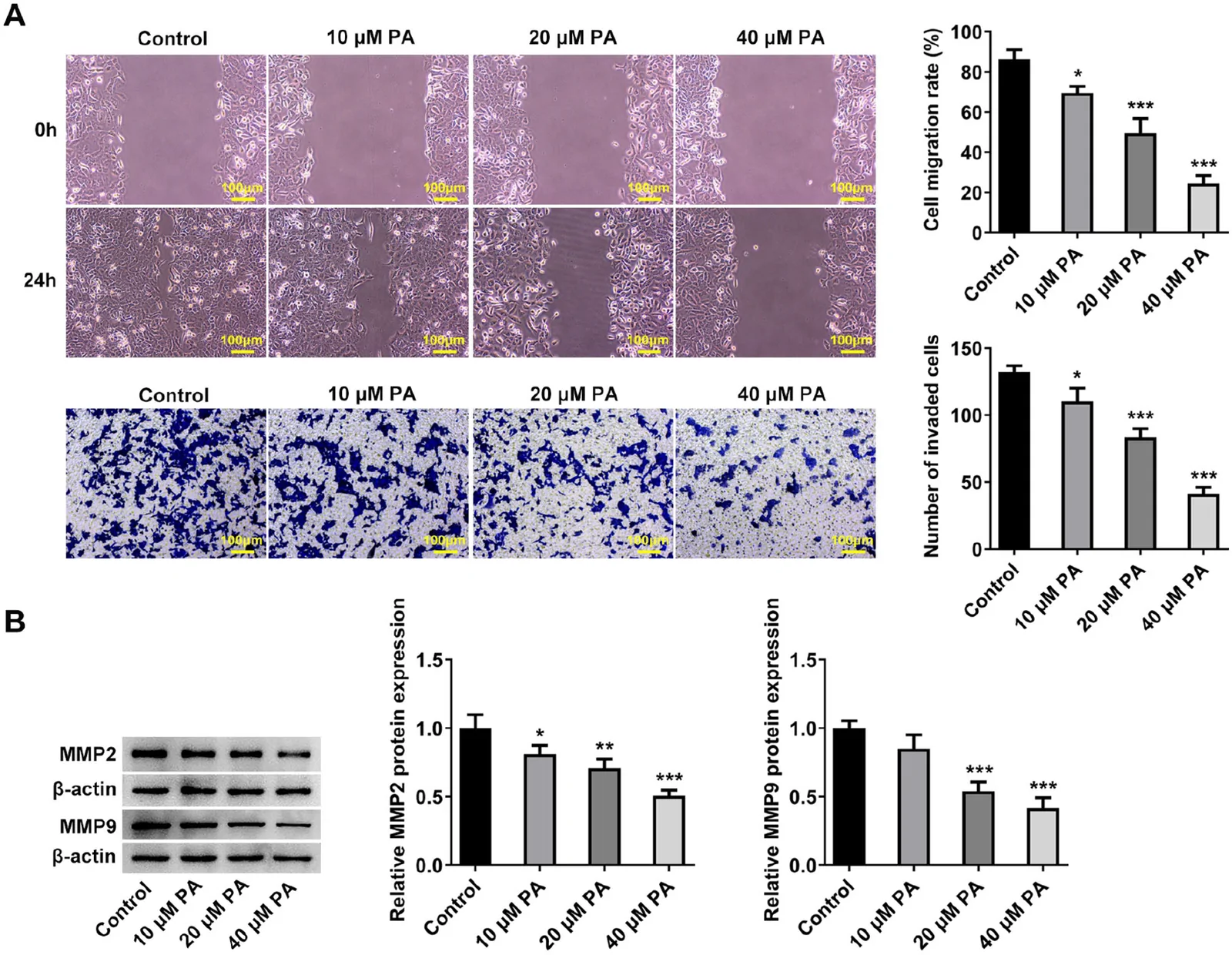
PA treatment inhibits the migration of CRC cells. **(A)** Wound healing assay (top) and transwell migration assay (bottom) showing dose-dependent inhibition of cell migration; quantification is shown in bar graphs. **(B)** Western blot analysis showing reduced MMP2 and MMP9 protein expression following PA treatment. Data are presented as mean ± SD. *p < 0.05, **p < 0.01, ***p < 0.001 vs control.

### PA induces mitochondria-mediated apoptosis in CRC cells

To delve deeper into the impact of PA on mitochondria-mediated apoptosis in CRC cells, we utilized DCFH-DA to quantify reactive oxygen species (ROS) levels within the cells. Furthermore, Rh123 staining was applied to monitor alterations in mitochondrial membrane potential. Additionally, western blot analysis was employed to detect the expression of proteins pertinent to apoptosis, specifically focusing on the distribution of Cytochrome C (Cyto C) and Bax between the mitochondria and cytoplasm. The results showed that with increasing PA concentrations, intracellular ROS levels rose significantly and mitochondrial membrane potential was progressively reduced in CRC cells (**Figures 3A, B**). In addition, with increasing PA concentrations, the expression of Bax, Bim, and cleaved-caspase3 was upregulated while Bcl-2 expression decreased. In the mitochondrial fraction, Cyto C decreased and Bax increased with increasing PA concentration; in the cytoplasmic fraction, Cyto C showed the opposite (reciprocal) pattern, increasing with PA treatment, consistent with its release from mitochondria. Cytoplasmic Bax also increased with PA treatment, in line with the overall upregulation of total Bax protein expression shown in **Figure 3C** (**Figures 3C, D**). The above data suggested that PA induced mitochondria-mediated apoptosis in CRC cells.

**Figure 3:**
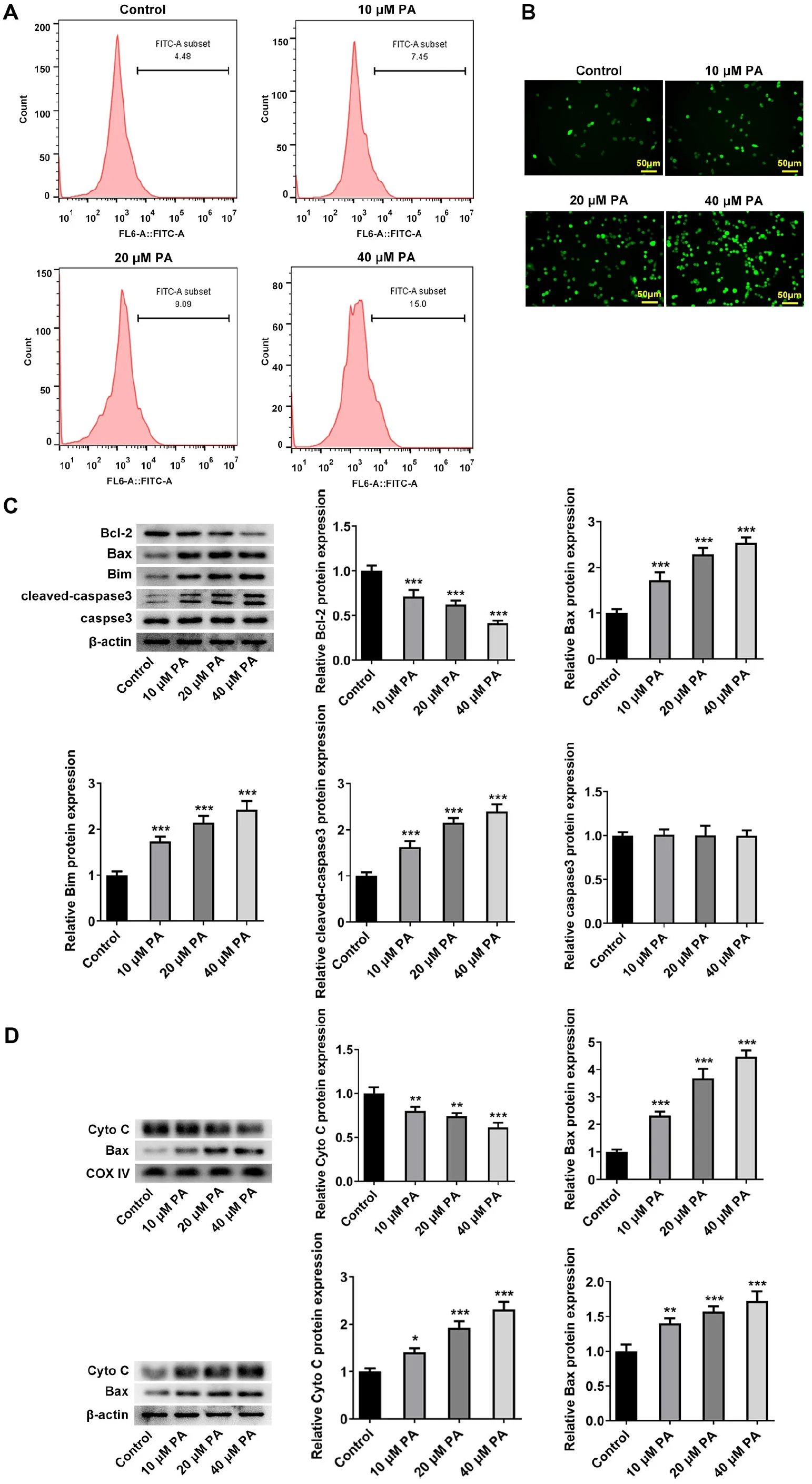
PA induces mitochondria-mediated apoptosis in CRC cells. **(A)** DCFH-DA fluorescence microscopy showing dose-dependent elevation of intracellular ROS levels. Fluorescence intensity was quantified using ImageJ. **(B)** Rh123 fluorescence imaging showing progressive mitochondrial membrane potential reduction. **(C)** Western blot analysis demonstrating upregulation of Bax, Bim, and cleaved-caspase3 and downregulation of Bcl-2 with increasing PA concentrations. **(D)** Western blot analysis of Cyto C and Bax distribution in mitochondrial and cytoplasmic fractions, showing Cyto C release from mitochondria to cytoplasm. Data are presented as mean ± SD. *p < 0.05, **p < 0.01, ***p < 0.001 vs control.

### PA down-regulates PTPN1 expression level in CRC cells

Subsequently, we investigated the influence of PA on the expression level of PTPN1 in CRC cells, employing qRT-PCR and western blot analysis to quantify PTPN1 mRNA and protein expression, respectively. Our findings indicated that PA led to a downregulation of PTPN1 expression in CRC cells (depicted in **Figure 4A**). Based on the outcomes of our preliminary experiments, a concentration of 40 μM PA was selected for subsequent investigative procedures. Then the three-dimensional structure of PTPN1 protein was obtained using the PDB database (https://www.rcsb.org/) (PDB ID: 1NWL), and molecular docking was performed using AutoDock. PA bound to PTPN1 with a predicted binding affinity of −6.3 kcal/mol, forming interactions with residues GLN262, ARG221, ILE219, LYS116, GLU115, LYS120, CYS47, and TYR46 within the active site (**Figure 4B**). To confirm PTPN1 overexpression, both qRT-PCR and western blot techniques were utilized. The results demonstrated successful PTPN1 overexpression in CRC cells (as shown in **Figure 4C**). Subsequently, these methods were employed again to quantify PTPN1 expression levels within the cells. Our data showed that compared with the PA+oe-NC group, the expression of PTPN1 in the PA+oe-PTPN1 group was significantly increased (**Figure 4D**).

**Figure 4:**
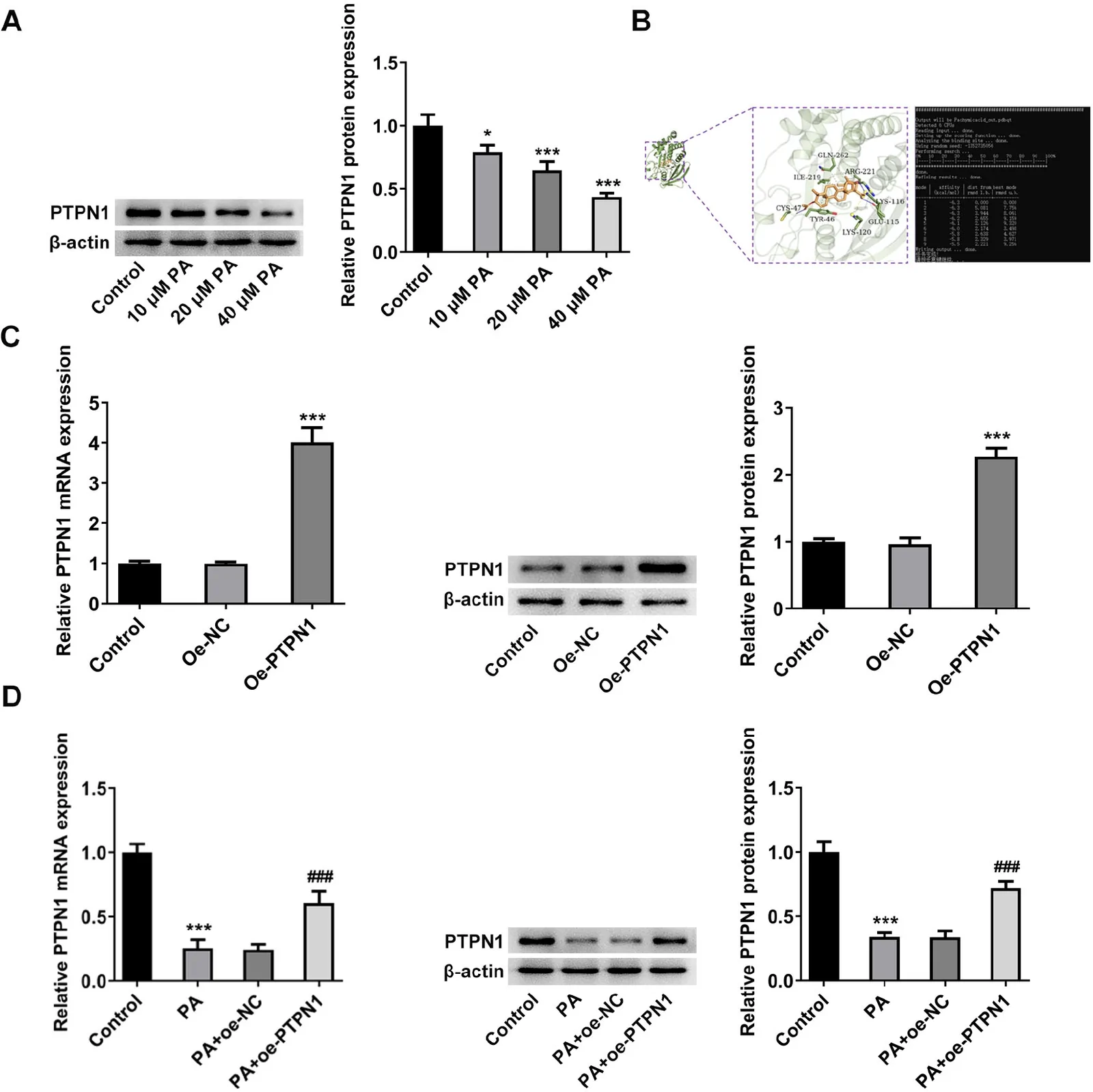
PA downregulates PTPN1 expression in CRC cells. **(A)** Western blot showing dose-dependent reduction in PTPN1 protein levels following PA treatment. **(B)** Three-dimensional structure of PTPN1 (PDB ID: 1NWL) and molecular docking result showing the predicted binding mode of PA at the PTPN1 active site (binding affinity: − 6.3 kcal/mol). **(C)** qRT-PCR and western blot confirming successful PTPN1 overexpression in HCT-116 cells, with Control, oe-NC, and oe-PTPN1 groups. **(D)** qRT-PCR and western blot showing that PTPN1 expression in the PA+oe-PTPN1 group was significantly higher than in the PA+oe-NC group. Data are presented as mean ± SD. *p < 0.05, ***p < 0.001 vs. control; ^###^p < 0.001 vs. PA+oe-NC.

### PA inhibits the proliferation and migration of CRC cells by downregulating PTPN1

To gain further insights into whether PA suppresses the proliferation and migration of CRC cells through the downregulation of PTPN1, we conducted CCK-8 and EdU staining assays to assess cellular proliferation rates. Additionally, a colony formation assay was performed to evaluate colony-forming capacity. Our results illustrated that overexpression of PTPN1 reversed the inhibition of PA on the proliferation and cellular colonies of CRC cells (**Figures 5A–C**). Subsequently, wound healing and transwell assays were used to detect cell migration, and western blot was used to detect the expression levels of migration-related proteins. The results showed that overexpression of PTPN1 reversed the inhibition of PA on CRC cell migration (**Figures 5D, E**). The above data revealed that PA inhibited the proliferation and migration of CRC cells by downregulating PTPN1.

**Figure 5:**
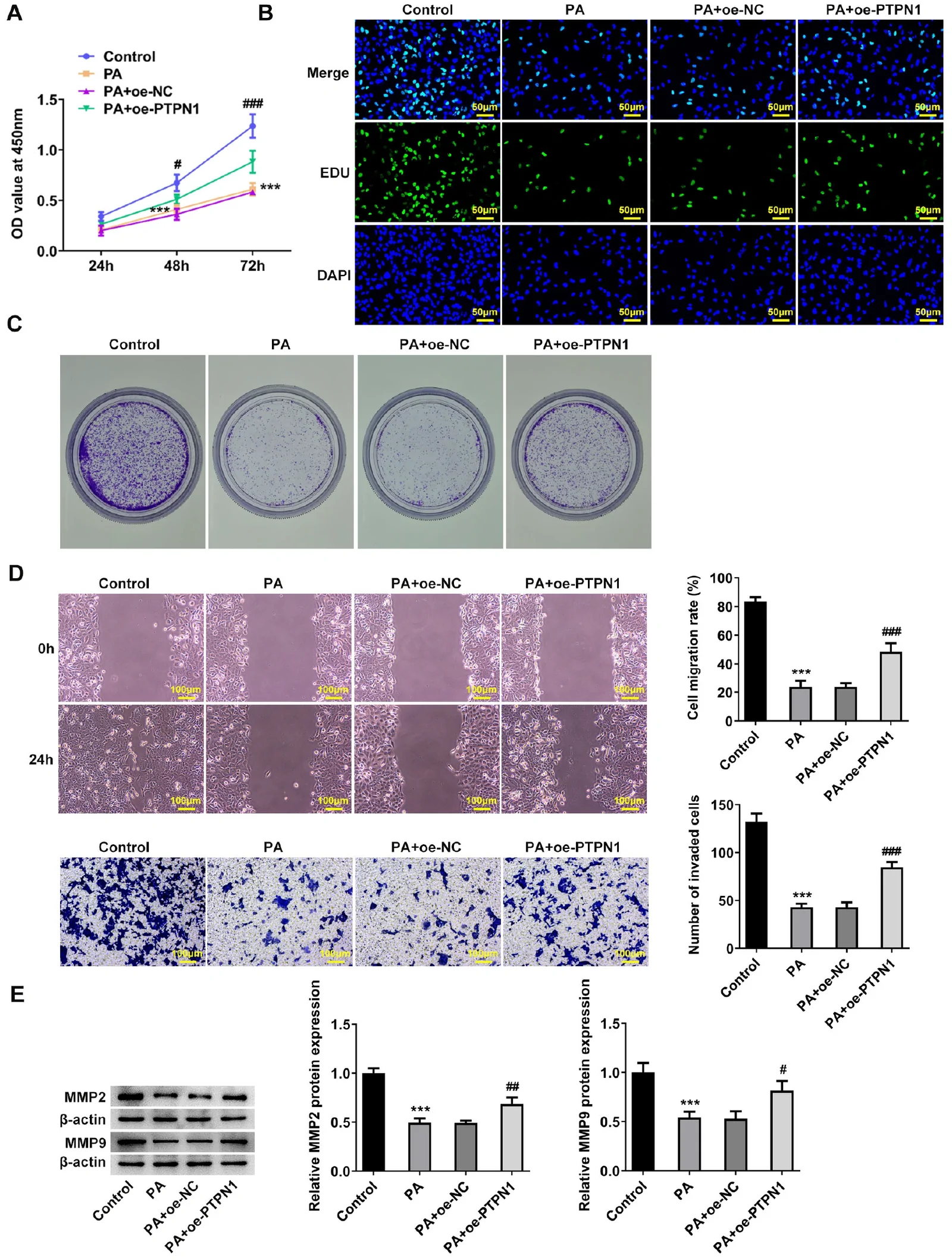
PA inhibits proliferation and migration of CRC cells by downregulating PTPN1. **(A)** CCK-8 assay showing that PTPN1 overexpression partially rescued the PA-induced inhibition of cell viability. **(B)** EdU staining showing increased DNA synthesis in PA+oe-PTPN1 cells compared to PA+oe-NC. **(C)** Colony formation assay showing partial restoration of colony formation by PTPN1 overexpression. **(D)** Wound healing (top) and transwell assay (bottom) demonstrating that PTPN1 overexpression reversed the PA-induced inhibition of migration; quantification shown in bar graphs. **(E)** Western blot showing partial restoration of MMP2 and MMP9 expression by PTPN1 overexpression. Data are presented as mean ± SD. ***p < 0.001 vs. control; ^#^p < 0.05, ^##^p < 0.01, ^###^p < 0.001 vs. PA+oe-NC.

### PA promotes mitochondria-mediated apoptosis of CRC cells by downregulating PTPN1

Subsequently, we investigated whether the downregulation of PTPN1 by PA enhances mitochondria-mediated apoptosis in CRC cells. To accomplish this, DCFH-DA was utilized to quantify the levels of ROS within the cells, and Rh123 staining was used to detect changes in mitochondrial membrane potential. The results showed that overexpression of PTPN1 reversed the elevation in ROS levels and the reduction in mitochondrial membrane potential caused by PA treatment (**Figures 6A, B**). Furthermore, western blot analysis was used to assess the expression of apoptosis-related proteins, as well as the subcellular distribution of Cytochrome C (Cyto C) and Bax between the mitochondria and cytoplasm. The findings indicated that overexpression of PTPN1 counteracted the effects of PA treatment on the expression of Bax, Bim, cleaved-caspase3, and Bcl-2 in CRC cells (**Figure 6C**). Additionally, in the mitochondrial-cytoplasmic fractionation assay, PTPN1 overexpression attenuated the PA-induced decrease in mitochondrial Cyto C and the corresponding increase in cytoplasmic Cyto C, and partially reversed the PA-induced increase in Bax in both fractions (**Figure 6D**). Overexpression of PTPN1 reversed the effects of PA treatment on apoptosis of CRC cells, and PA promoted mitochondria-mediated apoptosis of CRC cells by downregulating PTPN1.

**Figure 6:**
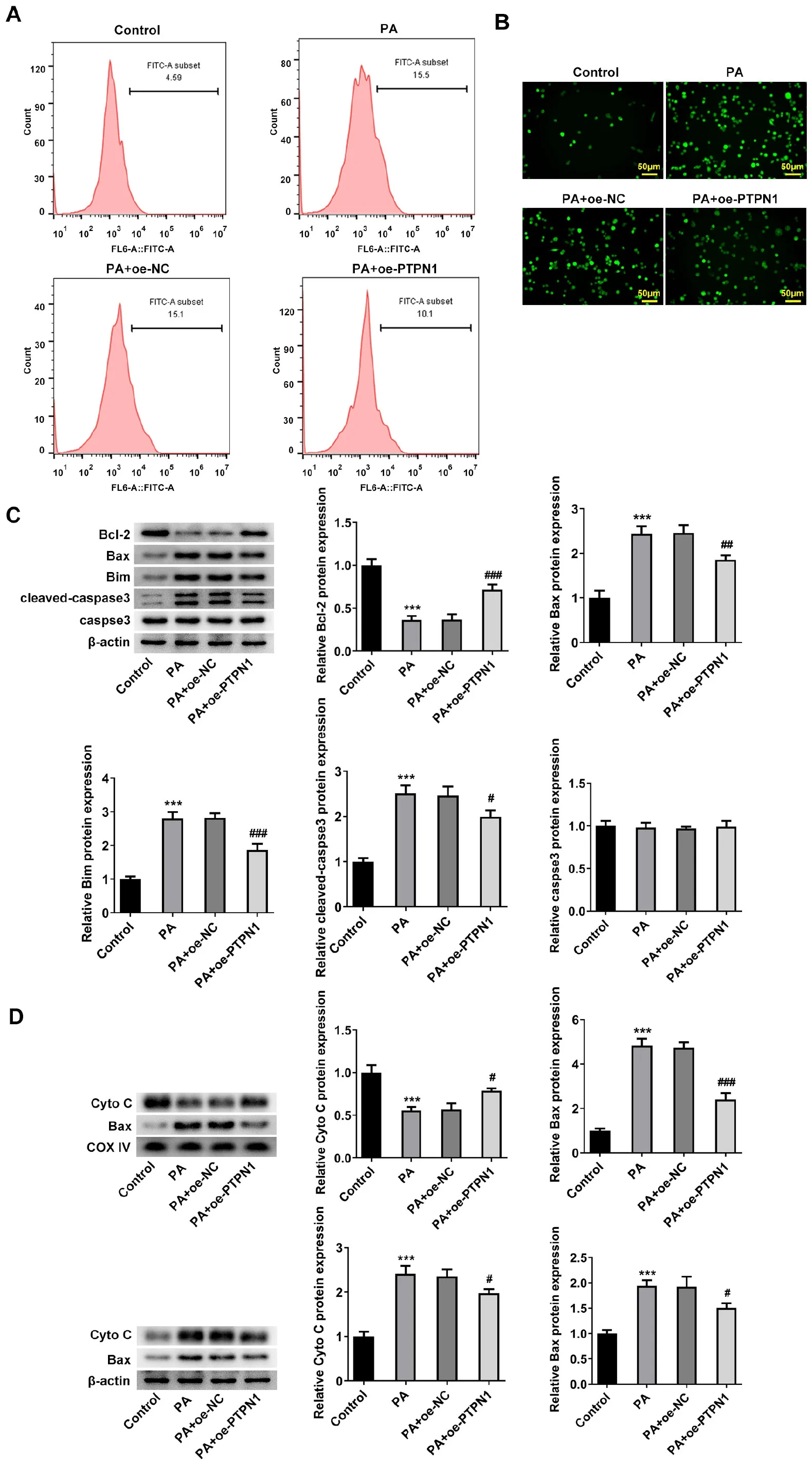
PA promotes mitochondria-mediated apoptosis in CRC cells by downregulating PTPN1. **(A)** DCFH-DA fluorescence microscopy showing that PTPN1 overexpression reduced PA-induced ROS accumulation. Fluorescence intensity was quantified using ImageJ. **(B)** Rh123 fluorescence imaging showing partial restoration of mitochondrial membrane potential by PTPN1 overexpression. **(C)** Western blot analysis showing that PTPN1 overexpression reversed the PA-induced changes in Bcl-2, Bax, Bim, and cleaved-caspase3 expression. **(D)** Western blot analysis of Cyto C and Bax in mitochondrial and cytoplasmic fractions, showing that PTPN1 overexpression attenuated PA-induced Cyto C release and Bax redistribution. Data are presented as mean ± SD. ***p < 0.001 vs. control; ^#^p < 0.05, ^##^p < 0.01, ^###^p < 0.001 vs. PA+oe-NC.

## Discussion

CRC persists as one of the leading causes of cancer-related deaths worldwide, with mortality rates remaining high for multiple decades (21, 22). Despite this alarming statistic, only a limited number of tumor markers have demonstrated clinical efficacy in the management of CRC (23). As such, there is a pressing need for the development of novel and promising therapeutic approaches. In our ongoing research, we have identified that PA has the potential to inhibit tumor progression in CRC cells by triggering mitochondria-mediated apoptosis through the downregulation of PTPN1.

PA, the predominant triterpenoid isolated from P. cocos (Schw.) Wolf, possesses pro-apoptotic, anti-inflammatory, and antitumor properties (13, 24). Various studies have highlighted PA’s ability to suppress the progression, invasion, and dissemination of tumor cells across different cancer types, including breast, lung, and pancreatic cancers (25–27). However, the literature on PA’s role and mechanisms in CRC treatment remains scant. Hence, the current investigation delved into the effects of PA on CRC cell proliferation and migration. Utilizing CCK-8 and EdU staining techniques, we observed a more pronounced inhibitory effect on CRC cell proliferation and colony formation with escalating PA concentrations. Furthermore, scratch healing, transwell migration, and western blot analyses demonstrated a dose-dependent reduction in MMP2 and MMP9 expression, alongside decreased cell migration following PA treatment.

Previous research has demonstrated that PA inhibits the tumorigenicity of gastric cancer cells through the mitochondrial apoptotic pathway (12). Furthermore, Li et al. have shown that PA activates the TP53INP2/TRAF6/caspase-8 pathway, thereby promoting apoptosis in renal cell carcinoma cells (10). In our current study, we aimed to further elucidate the impact of PA on mitochondrial apoptosis in CRC cells. Our findings revealed that as the concentration of PA treatment increased, there was a significant elevation in ROS levels and mitochondrial membrane potential reduction in CRC cells. Additionally, we observed a notable upregulation in the expression of Bax, Bim, and cleaved-caspase3, accompanied by a downregulation in Bcl-2 expression. Furthermore, with increasing PA concentrations, the expression of Cyto C in the mitochondria of CRC cells decreased, while the expression of Bax increased. In the cytoplasm, Cyto C increased, consistent with its release from mitochondria. Cytoplasmic Bax also increased, in line with the overall upregulation of total Bax protein expression. Collectively, these data suggest that PA induces mitochondria-mediated apoptosis in CRC cells.

PTPN1, a prominent member of the PTPN family, functions as a crucial negative regulator of insulin and leptin receptor signaling pathways (28, 29). Recent studies indicate that PTPN1’s role is context-dependent, exhibiting both tumor suppressive and tumor promotional activities across different tumor types (30). However, existing research on PTPN1 is confined to a select few tumor types, and its prognostic significance and precise role in human tumors remain largely unknown and warrant further exploration (31). In our current study, we investigated the impact of PA on PTPN1 expression levels in CRC cells. Our findings revealed that PA downregulated PTPN1 expression in these cells. Based on our experimental outcomes, 40 μM PA was chosen for subsequent experiments. The successful overexpression of PTPN1 in CRC cells was confirmed through qRT-PCR and western blot analyses. Furthermore, we observed that compared to the PA+oe-NC group, the PA+oe-PTPN1 group exhibited a significant increase in PTPN1 expression.

Mechanistically, PTPN1/PTP1B is known to dephosphorylate and thereby attenuate signaling through several receptor tyrosine kinases and downstream effectors, including the PI3K/Akt and STAT3 pathways, both of which converge on the transcriptional and post-translational regulation of Bcl-2 family proteins. We speculate that PA-induced PTPN1 downregulation may relieve this negative regulation only partially, or alternatively act through a PTPN1-dependent but Akt/STAT3-independent route; the precise signaling intermediates linking PTPN1 downregulation to Bcl-2/Bax rebalancing were not directly examined here and represent an important direction for future work.

We then delved into whether PA inhibits the proliferation and migration of CRC cells by downregulating PTPN1. Our results demonstrated that PTPN1 overexpression could counteract the inhibitory effects of PA on these cellular processes. In summary, these data suggest that PA inhibits the proliferation and migration of CRC cells by downregulating PTPN1. Subsequently, we examined whether the downregulation of PTPN1 by PA enhances mitochondria-mediated apoptosis in CRC cells. Our findings indicated that PTPN1 overexpression could counteract the elevation in ROS levels and the alteration in mitochondrial membrane potential that were induced by PA treatment. Furthermore, PTPN1 overexpression reversed the modulatory effects of PA on the expression levels of Bax, Bim, cleaved caspase-3, and Bcl-2 in CRC cells, as well as the distribution of Cyto C and Bax between the cytoplasm and mitochondria. Notably, PTPN1 overexpression mitigated the apoptotic effects of PA treatment on CRC cells, suggesting that PA promoted mitochondria-mediated apoptosis in CRC cells through the downregulation of PTPN1.

An important consideration for the translational relevance of this study is whether the 40 μM PA concentration used in our mechanistic experiments is achievable *in vivo*. Based on the molecular weight of PA (528.76 g/mol), 40 μM corresponds to approximately 21.2 μg/mL. Published pharmacokinetic data show that after a single oral dose of PA alone (10 mg/kg) in rats, the peak plasma concentration (Cmax) was 333.4 ± 138.8 ng/mL (approximately 0.63 μM), with an elimination half-life (t1/2β) of 4.96 ± 1.33 h and an AUC0→∞ of 1466.9 ± 361.7 ng·h/mL (32). This indicates that the concentration used in our *in vitro* experiments substantially exceeds the systemic exposure achievable after a single oral dose of PA alone. However, two points warrant consideration. First, CRC is a gastrointestinal tumor directly exposed to luminal drug concentrations after oral administration, which may be substantially higher than systemic plasma levels; whether locally achievable colonic concentrations approach the range used here remains to be directly tested. Second, PA is typically administered as part of Poria cocos extract or Jianpi Jiedu Decoction rather than as an isolated compound, and repeated or combined dosing, or formulation strategies to improve bioavailability, may allow higher sustained exposure than reported after a single dose of PA alone. Direct pharmacokinetic and tissue-distribution studies of PA in the colon, together with *in vivo* efficacy testing at clinically achievable doses, will be required to establish the clinical relevance of the concentrations used in this study.

Several limitations of this study should be acknowledged. First, all experiments were conducted in a single CRC cell line (HCT-116); due to current funding constraints, validation in additional CRC cell lines with different genetic backgrounds could not be completed in this study, although HCT-116 is a well-characterized model widely used in comparable mechanistic studies. Future work incorporating additional cell lines (e.g., SW480, HT-29) will be needed to confirm the generalizability of our findings. Second, the cytotoxic profile of PA in normal colonic epithelial cells (e.g., NCM460 or CCD 841 CoN) was not assessed in this study due to resource constraints; consequently, whether PA exerts cancer-selective activity in the colorectal context, as opposed to general cytotoxicity, remains to be established, and this represents an important priority for follow-up work. Third, *in vivo* studies using xenograft mouse models are needed to confirm the antitumor efficacy of PA in a physiological context, together with direct pharmacokinetic and tissue-distribution data as discussed above. Fourth, the precise upstream mechanism by which PA downregulates PTPN1 at the transcriptional or post-translational level remains to be determined. Fifth, although molecular docking and PTPN1 overexpression rescue experiments support a functional role for PTPN1 in PA’s mechanism of action, direct target-engagement evidence (e.g., CETSA, SPR, or DARTS) was not obtained, and the conclusion should therefore be interpreted as PTPN1 being functionally involved in, rather than unequivocally the direct binding target of, PA. Sixth, this study relied solely on a PTPN1 overexpression rescue strategy to demonstrate the functional involvement of PTPN1; a complementary loss-of-function approach (e.g., PTPN1 knockdown or knockout in the absence of PA) would further strengthen the causal claim that PTPN1 downregulation alone is sufficient to phenocopy PA’s proapoptotic and antimigratory effects, and should be included in future studies. Future studies addressing these points will be critical for moving toward preclinical development of PA as an anticancer agent.

In conclusion, PA inhibited CRC cell proliferation and migration, induced mitochondria-mediated apoptosis through ROS accumulation and regulation of Bcl-2 family proteins, and downregulated PTPN1 expression. PTPN1 overexpression reversed all of the above effects, indicating that PTPN1 is functionally involved in the antitumor action of PA in CRC cells. These findings provide mechanistic insights into the PTPN1 dependent pathway of PA *in vitro* anti-tumor activity, representing preliminary mechanistic findings in HCT-116 CRC cells. Before evaluating its translational or therapeutic value, further assessment of normal cell safety, pharmacokinetics, and *in vivo* therapeutic efficacy is needed.

## Data Availability

The original contributions presented in the study are included in the article/**Supplementary Material**. Further inquiries can be directed to the corresponding authors.
