# Supplementary material for: PTPN1 downregulation contributes to pachymic acid-induced mitochondrial apoptosis in colorectal cancer cells
Source: Front Oncol. 2026 Oct 13;16:1891182. doi: 10.3389/fonc.2026.1891182 (PMC13710042; doi:10.3389/fonc.2026.1891182)

Pachymic acid inhibits colorectal cancer cell proliferation and migration by downregulating PTPN1 and inducing mitochondria-mediated apoptosis

Chuang Liu[1, 2†], Qiang Su[1†], Zhenxiang An[1, 2], Sufang Zhou[1, 2*], Wen Che[3*]

[1]The First Clinical Medical College, Guizhou University of Traditional Chinese Medicine, Guiyang, China.

[2]Department of Gastroenterology, First Affiliated Hospital of Guizhou University of Traditional Chinese Medicine, Guiyang, China.

[3]Department of Pathology, Ruijin Hospital, Shanghai Jiaotong University School of Medicine, Shanghai 200025, China.

[*]CORRESPONDENCE: Wen Che: Wenche197@163.com

Sufang Zhou: 1416439978@qq.com

[†]Both of these authors made equal contributions to this study and share the position of first author.

The original and uncropped membrane blots

The bands of the reference protein (beta-actin or GAPDH) used as loading control were from the same blot as the test protein(s). They were showed as follow：

Fig2B

MMP2

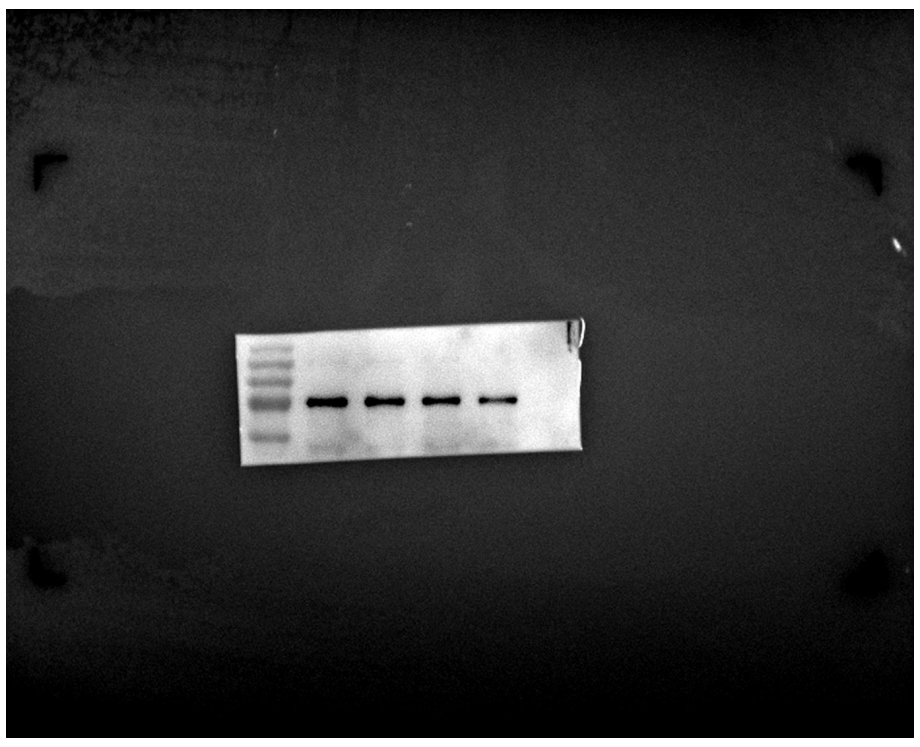

MMP9

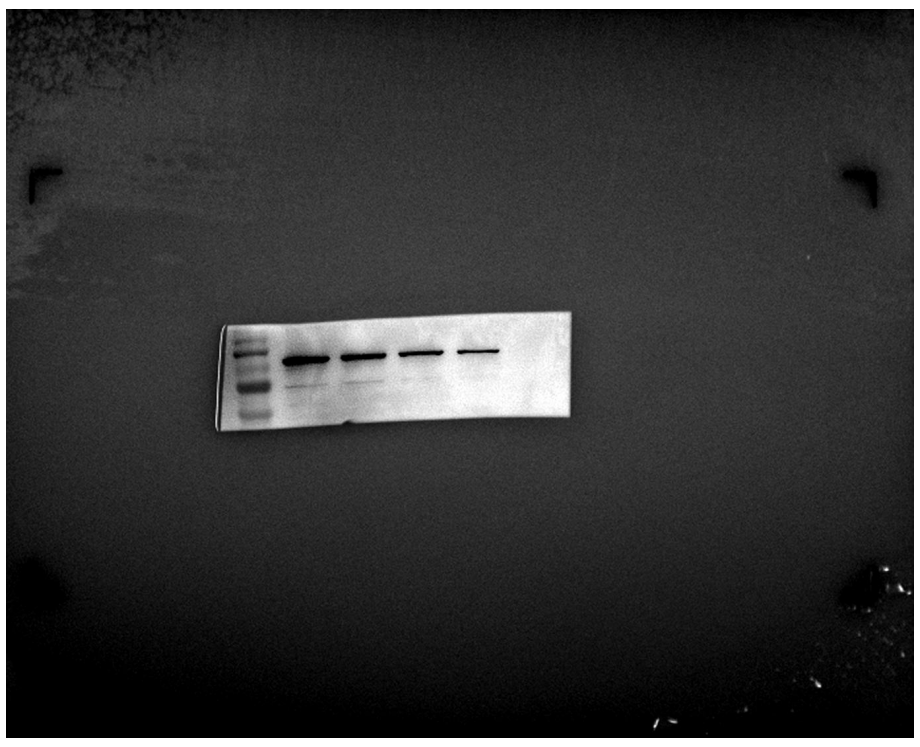

β-actin

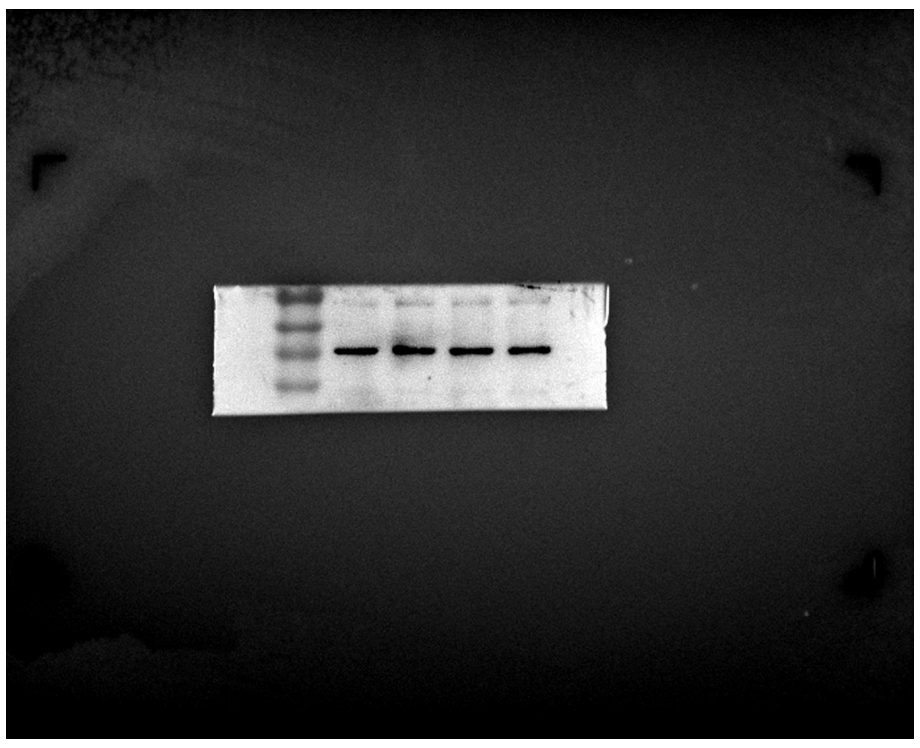

Fig3C

Bcl-2

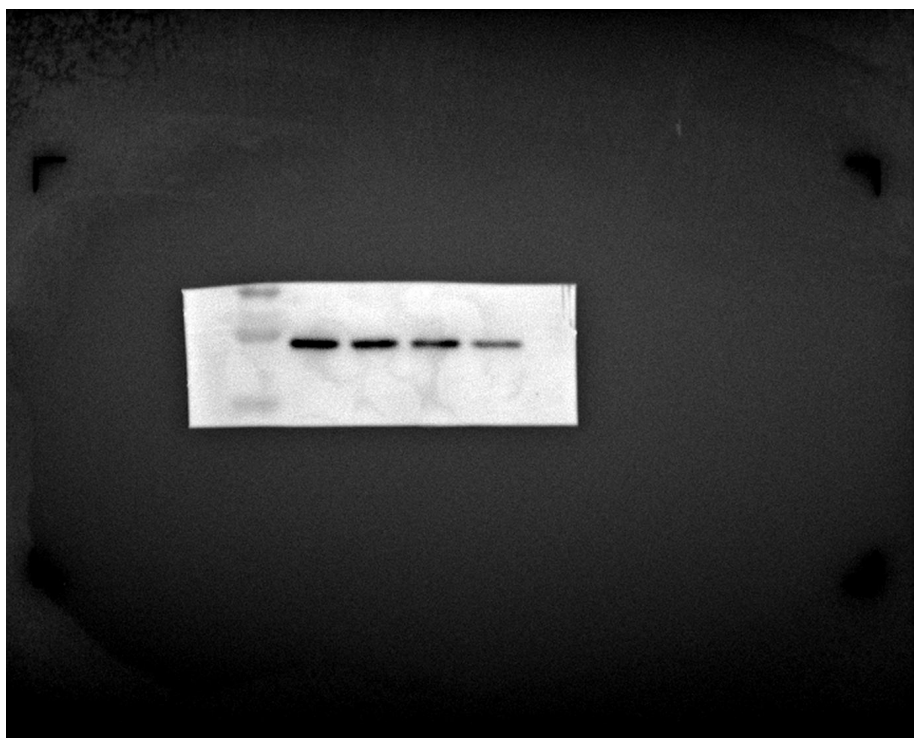

Bax

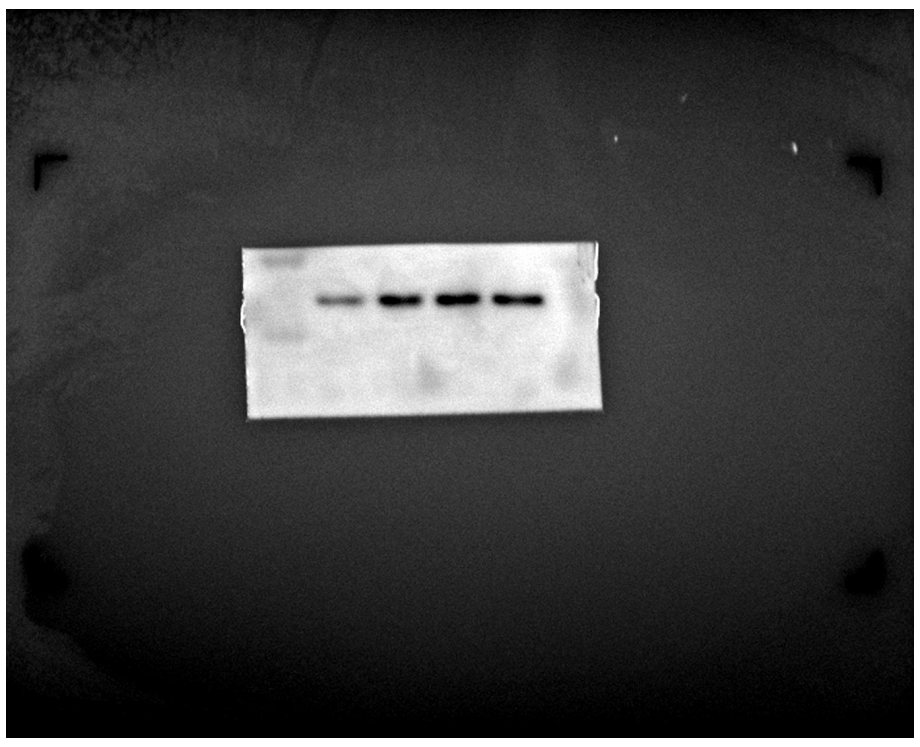

Bim

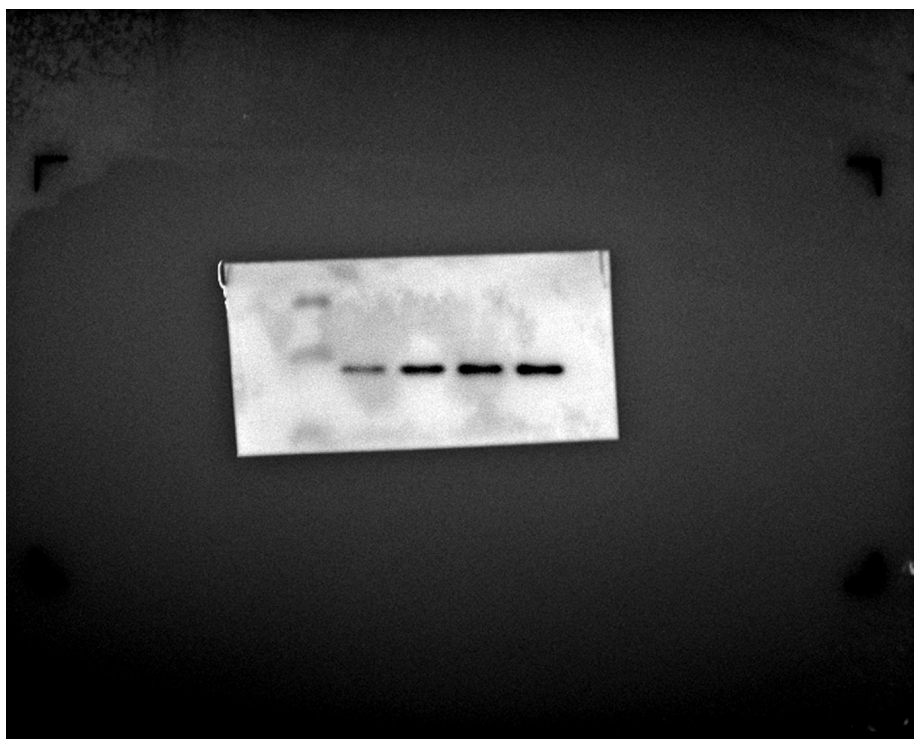

cleaved-caspase3

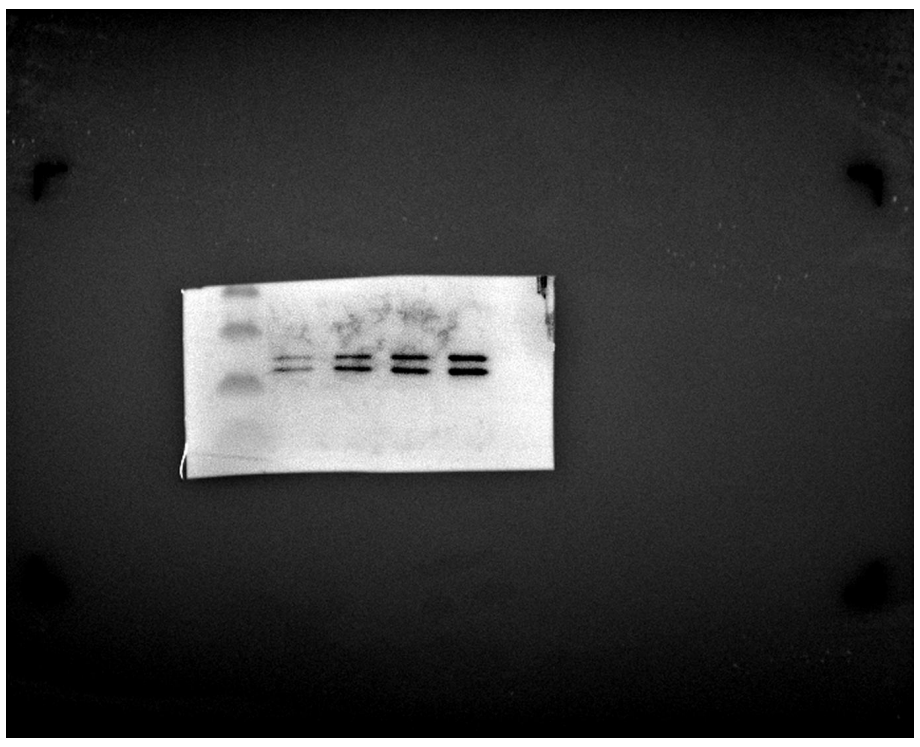

caspase3

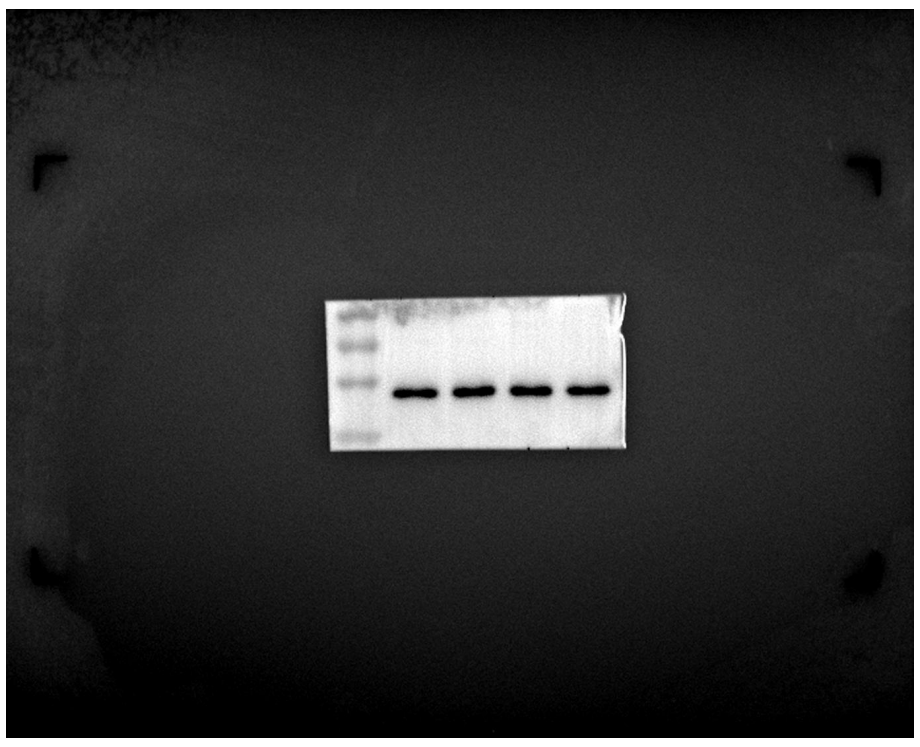

β-actin

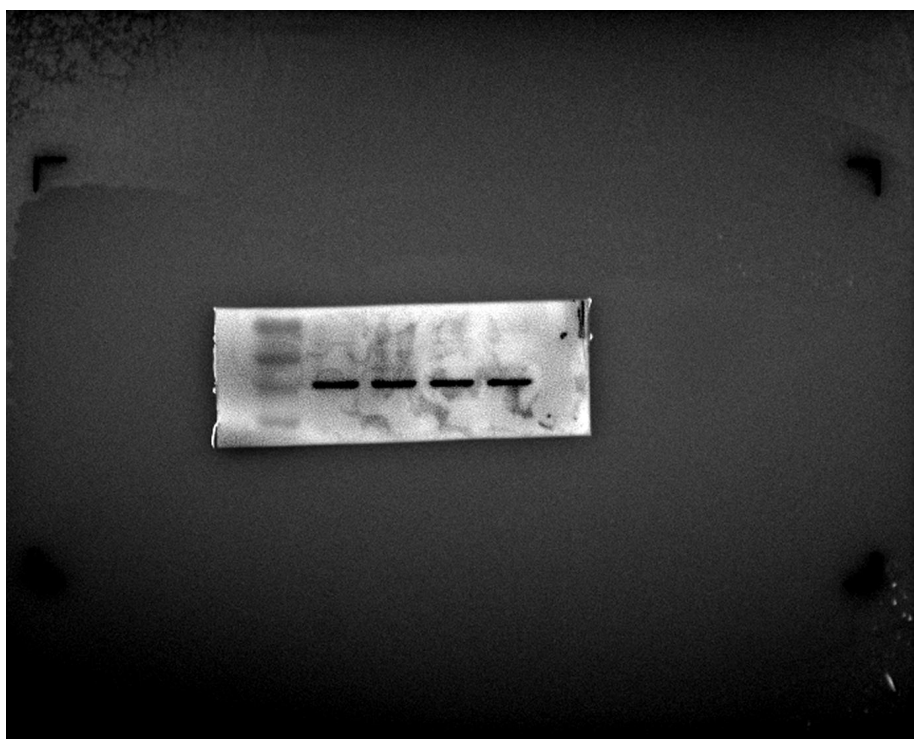

Fig3D

Cyto C

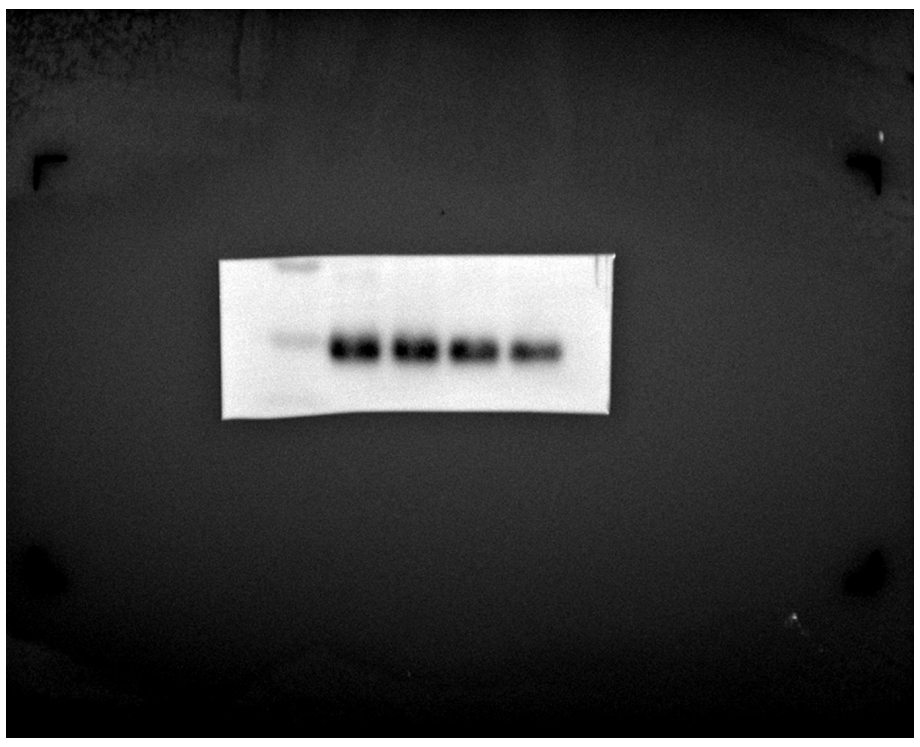

Bax

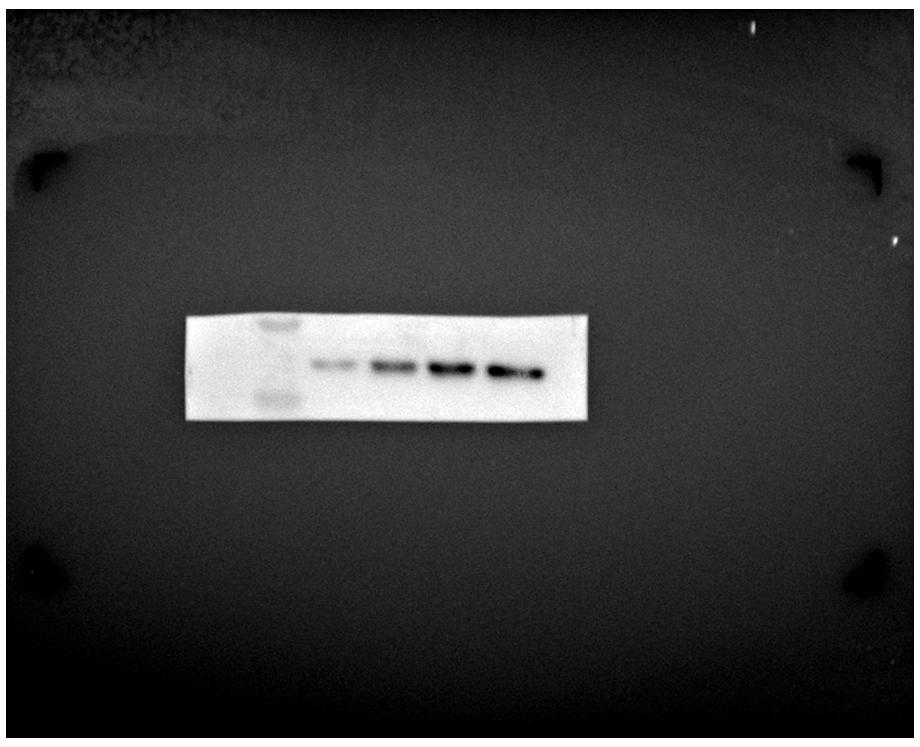

COX IV

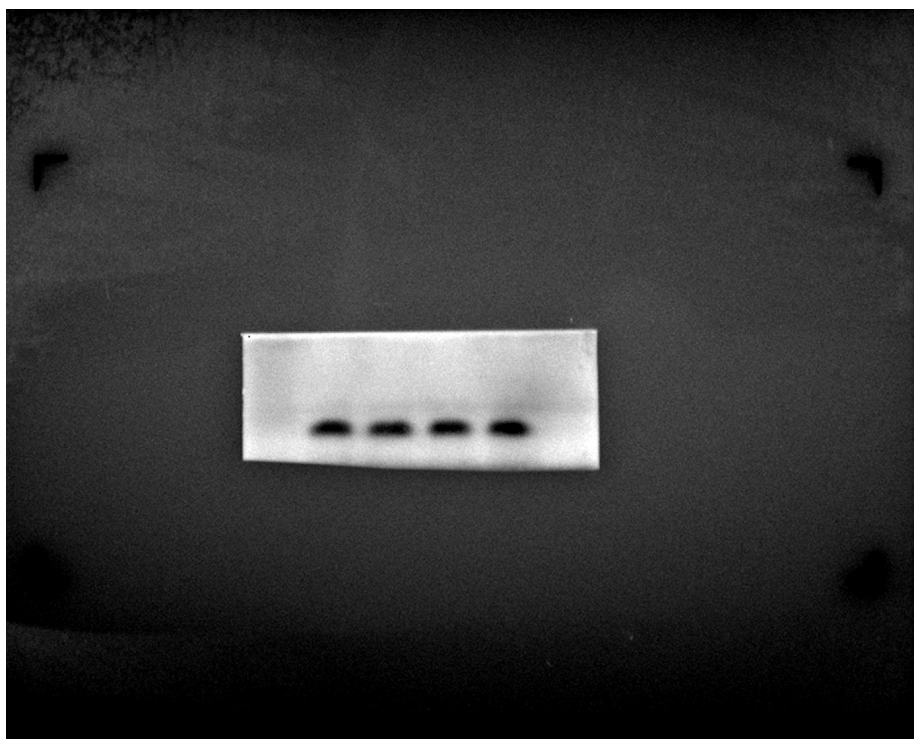

Cyto C

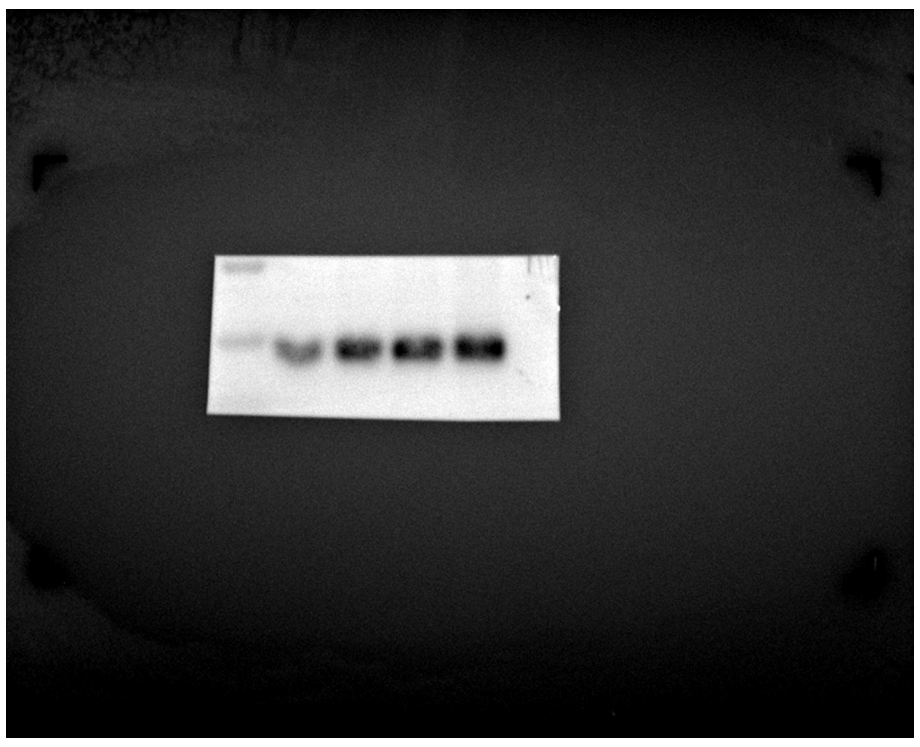

Bax

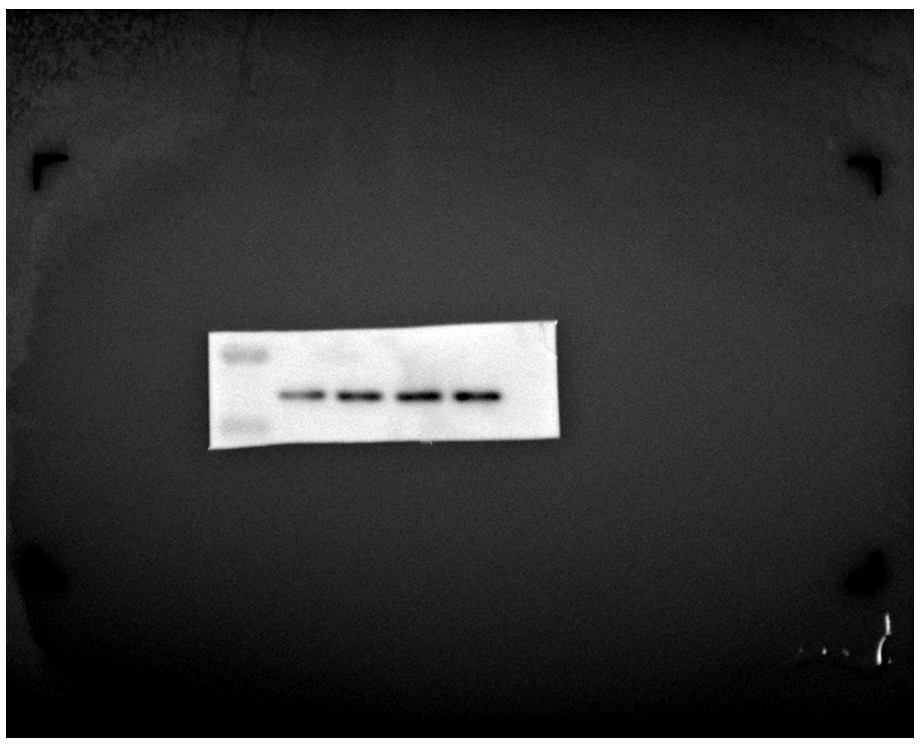

β-actin

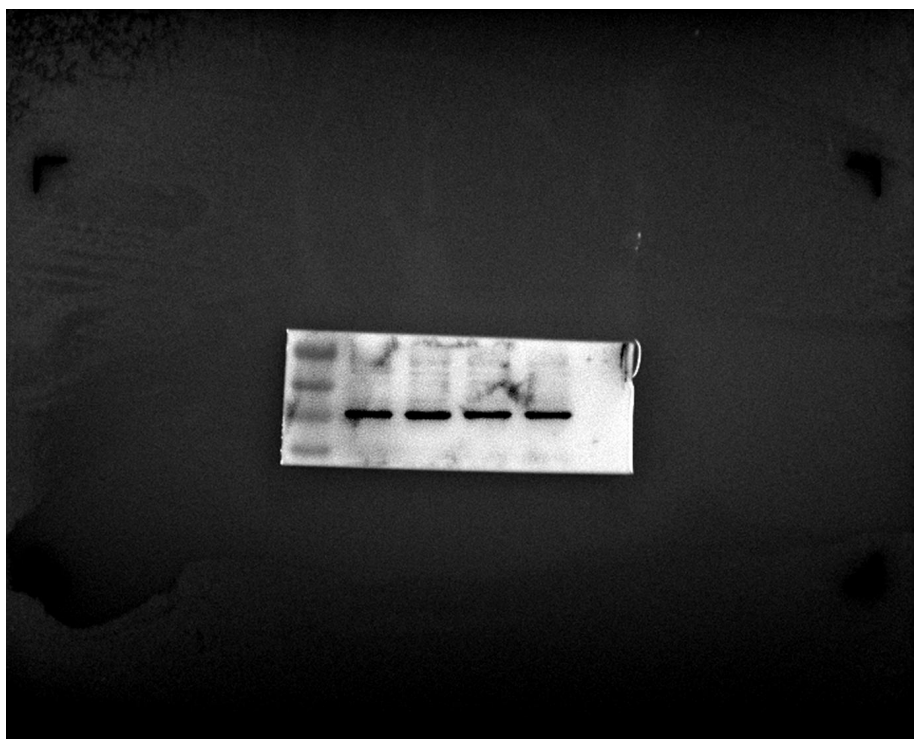

Fig4A

PTPN1

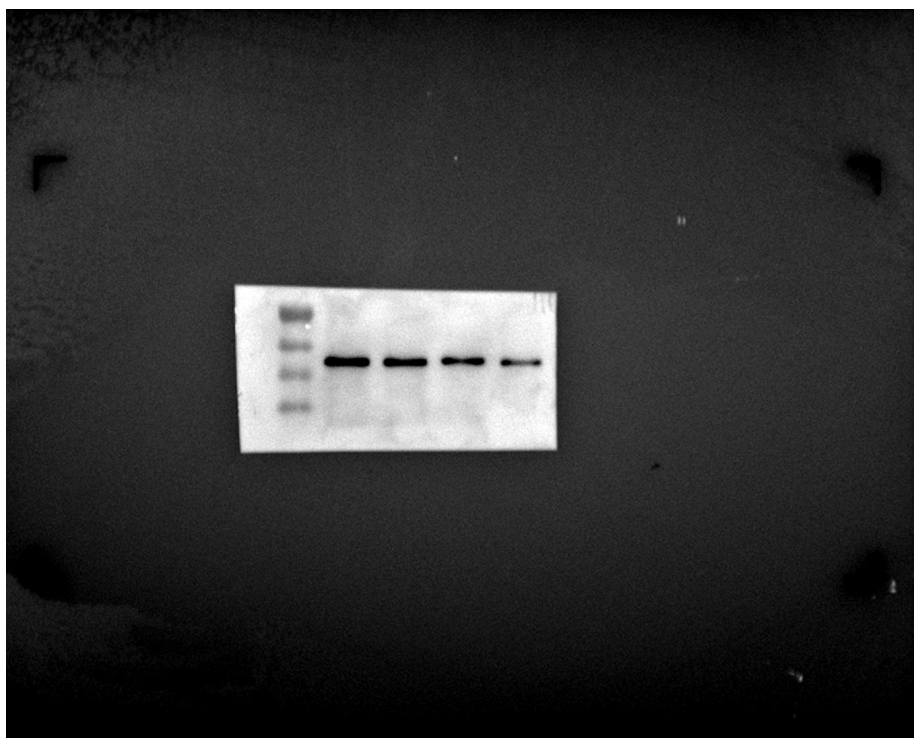

β-actin

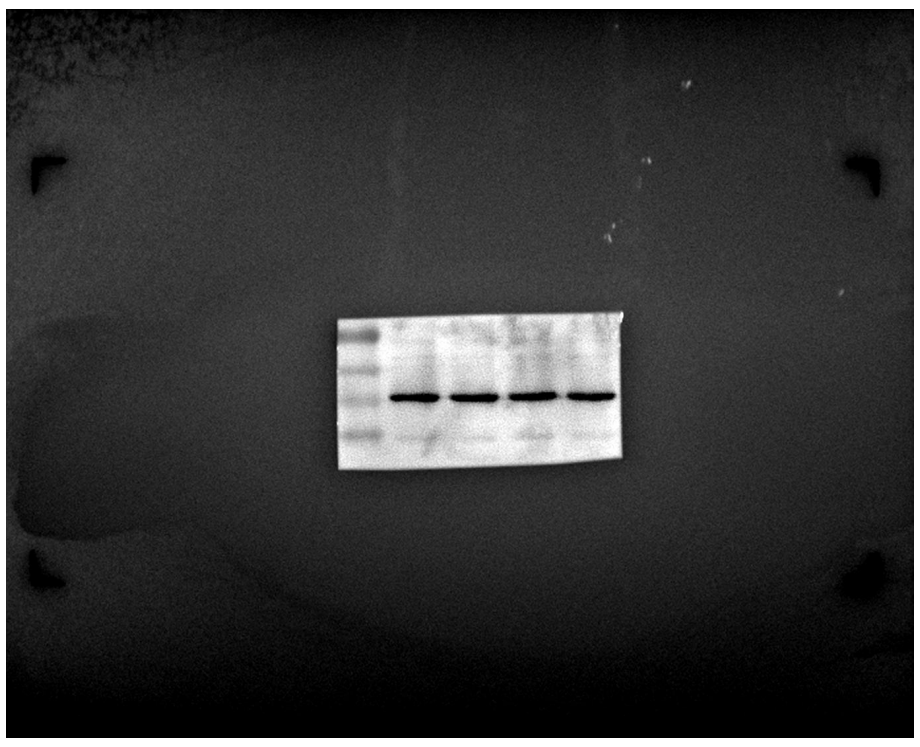

Fig4C

PTPN1

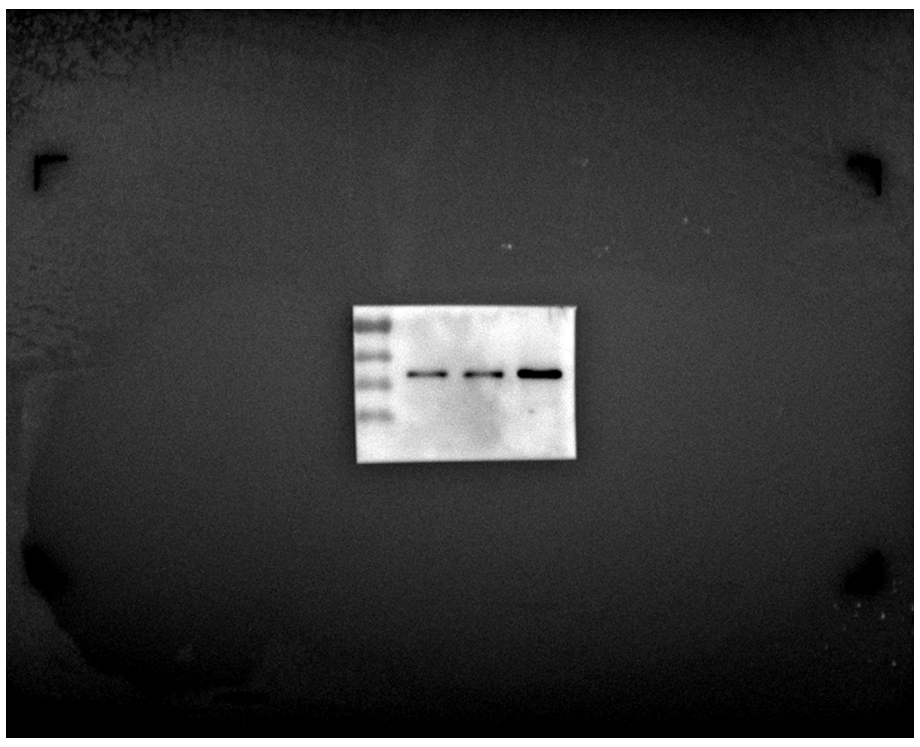

β-actin

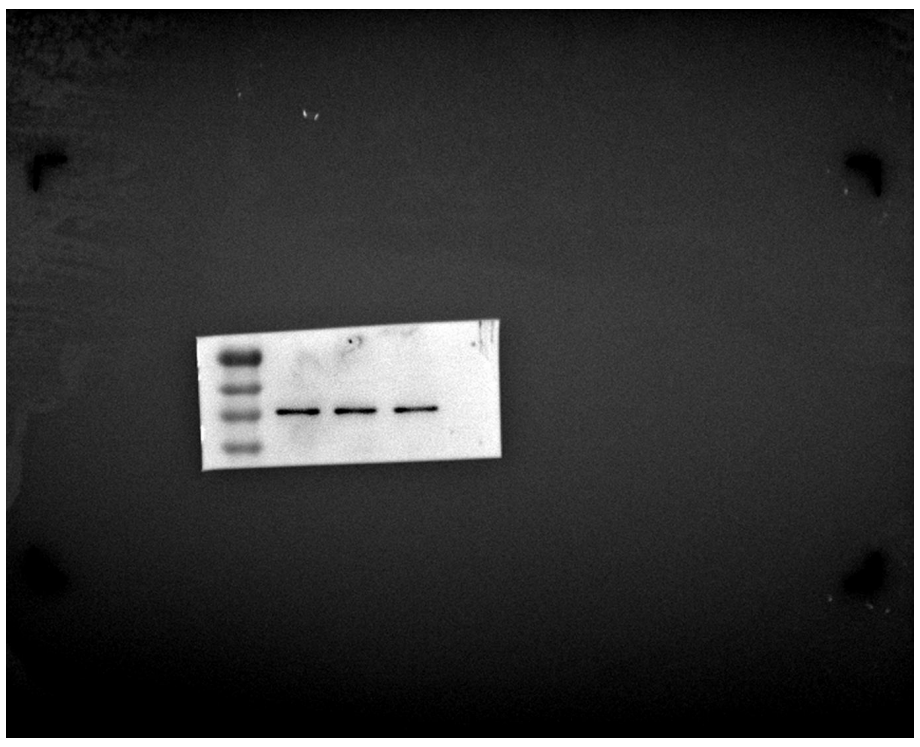

Fig4C

PTPN1

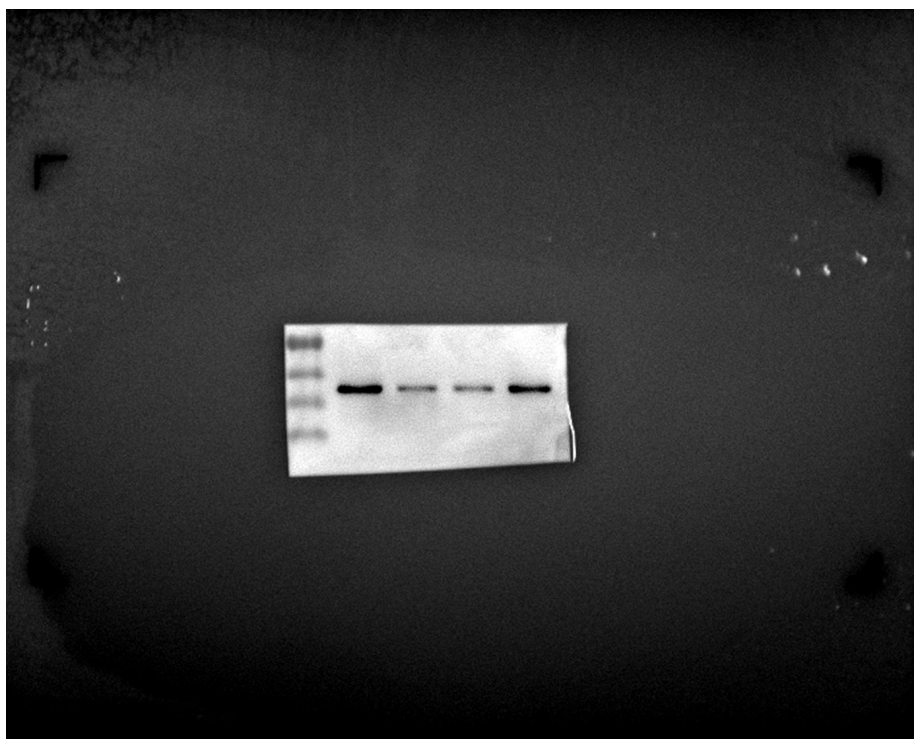

β-actin

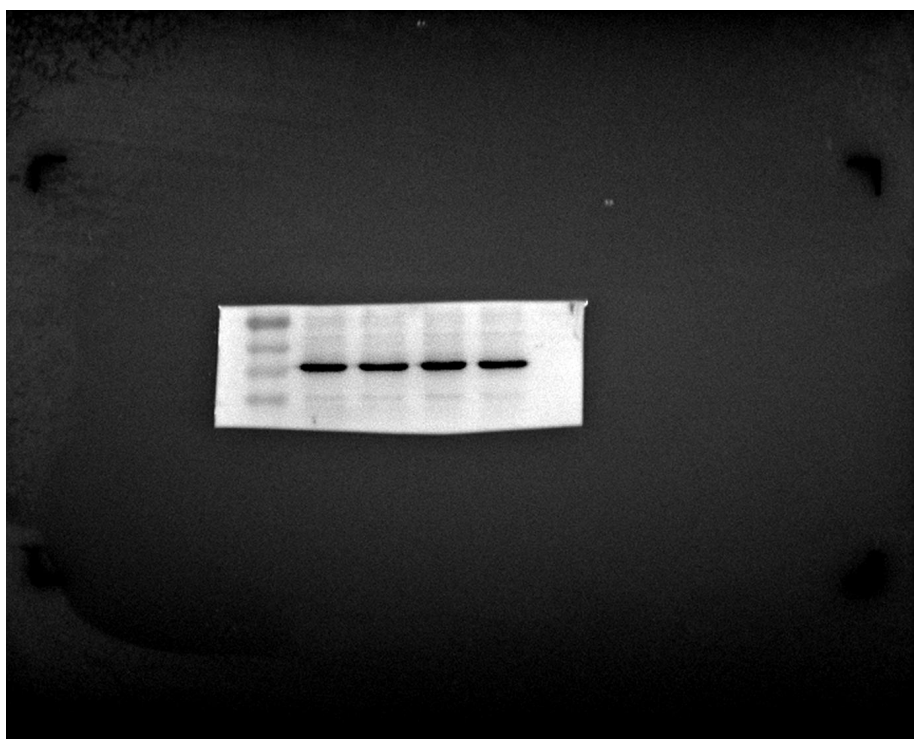

Fig5E

MMP2

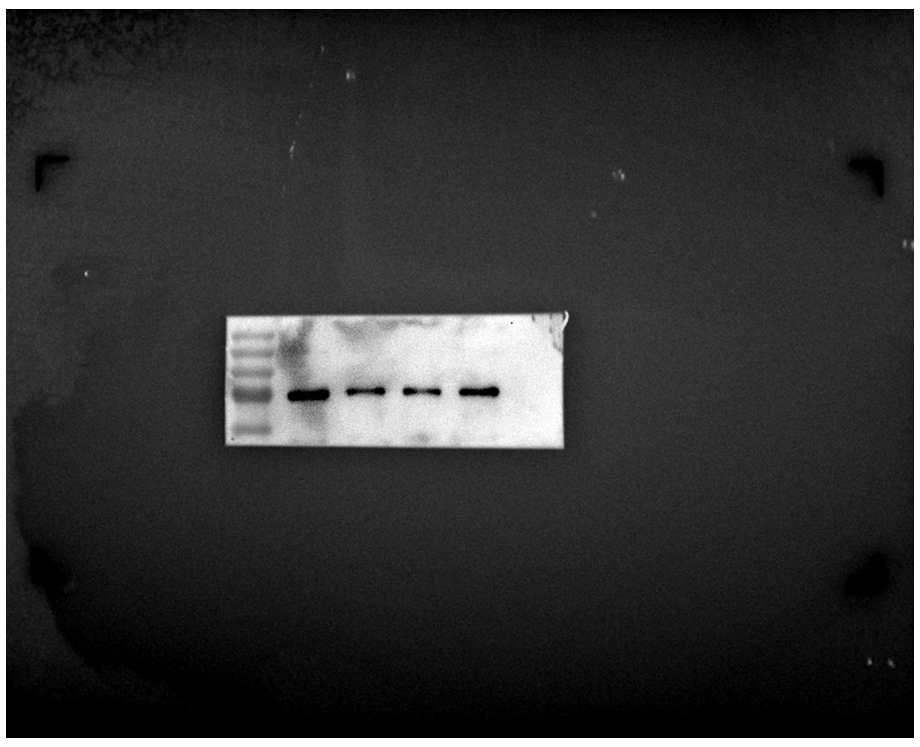

β-actin

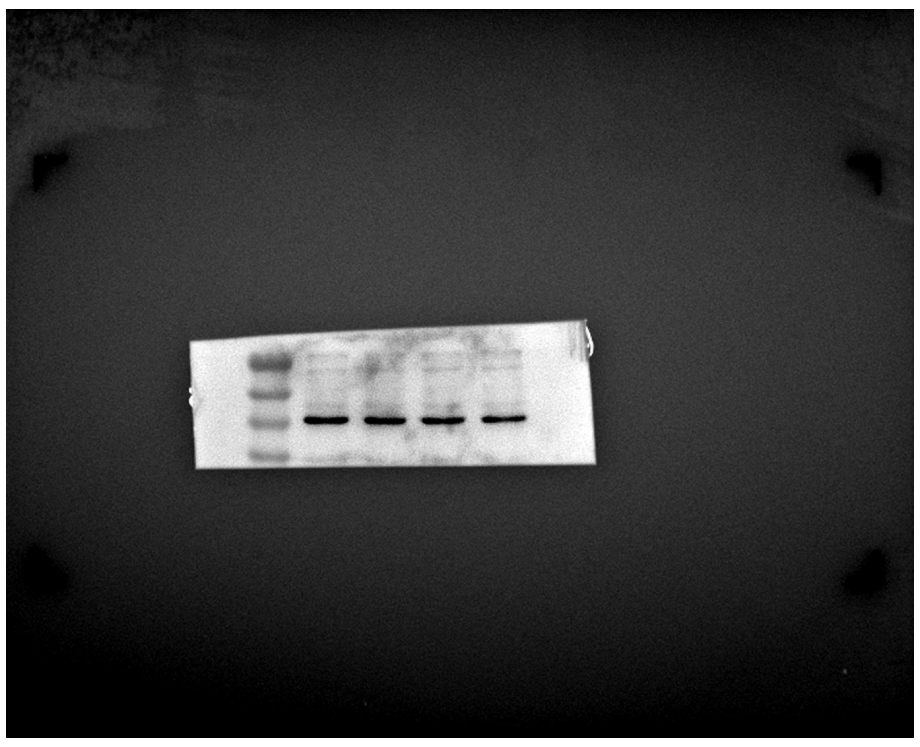

MMP9

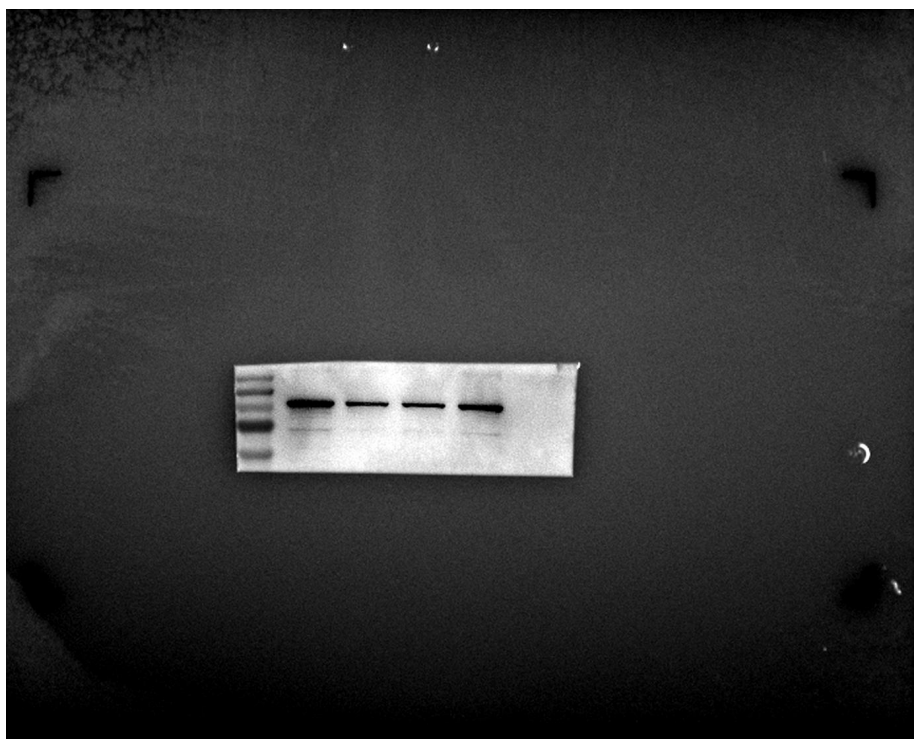

β-actin

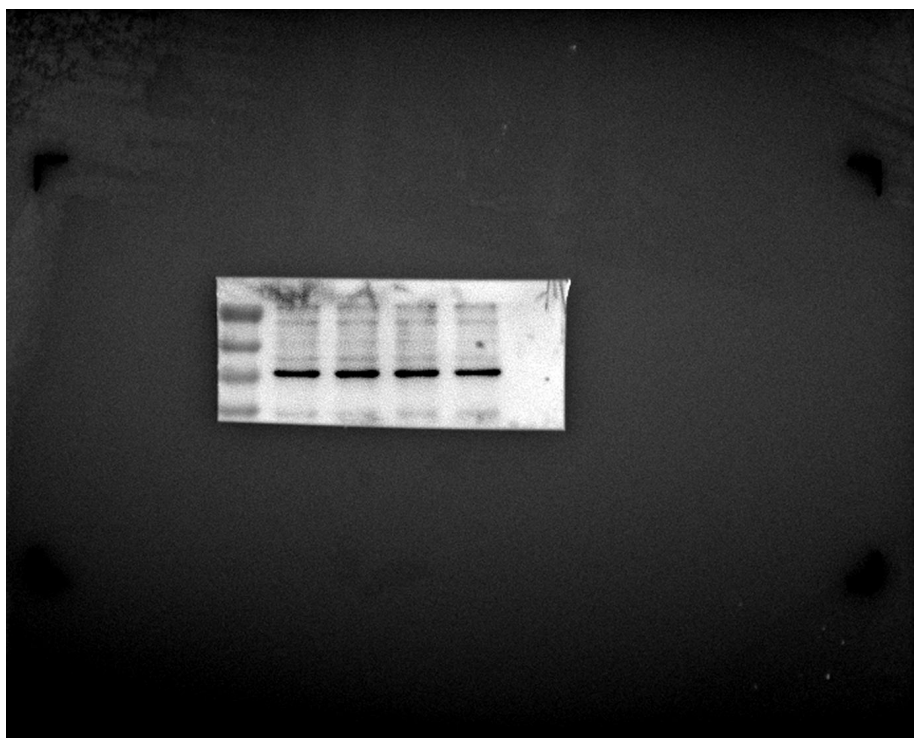

Fig6C

Bcl-2

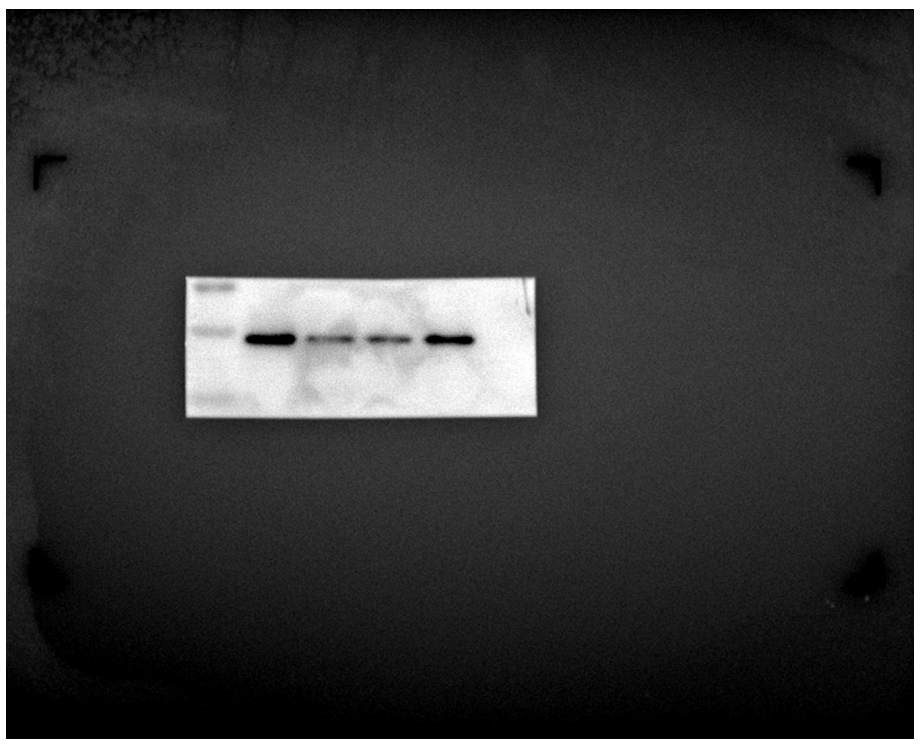

Bax

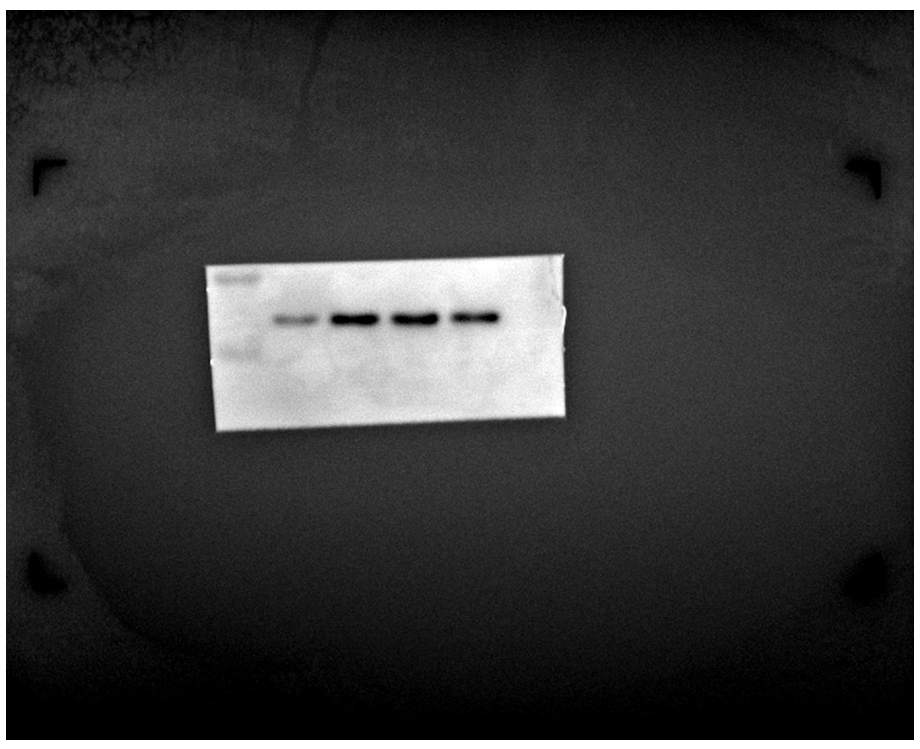

Bim

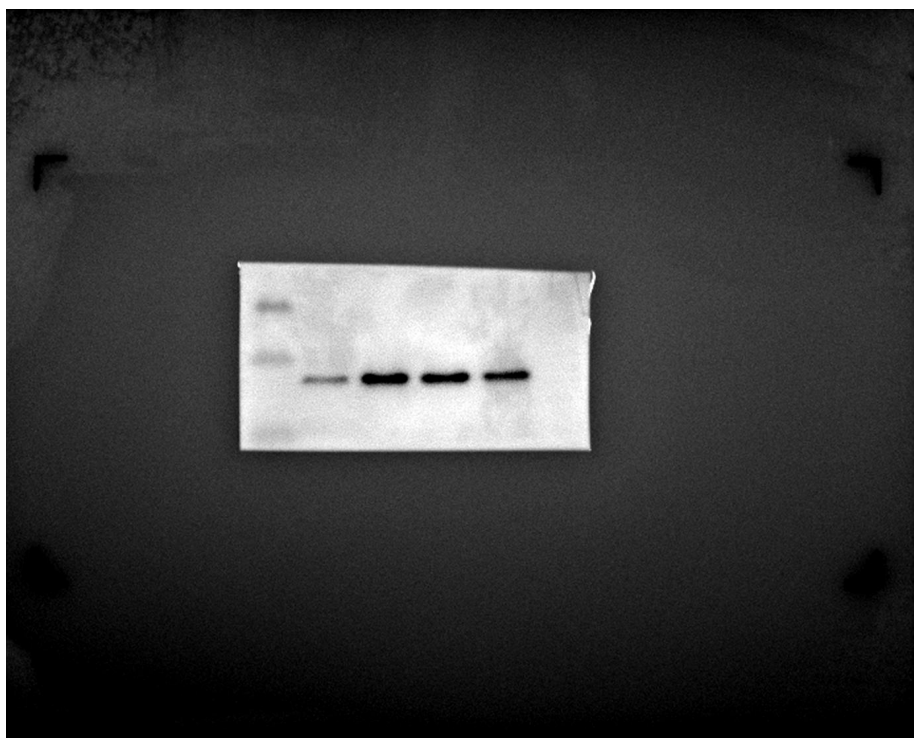

cleaved-caspase3

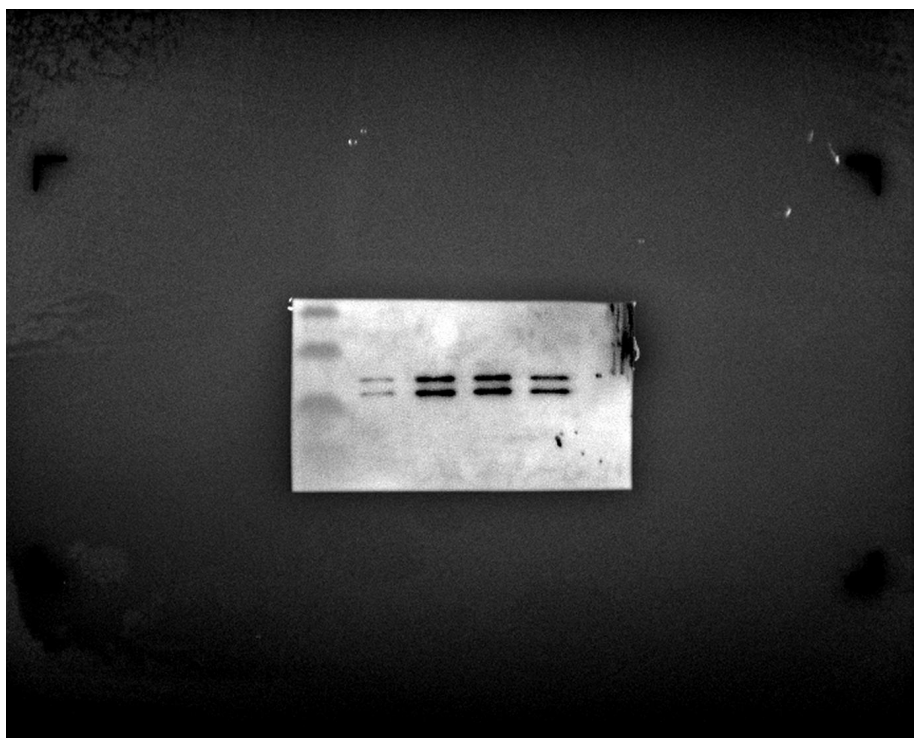

caspase3

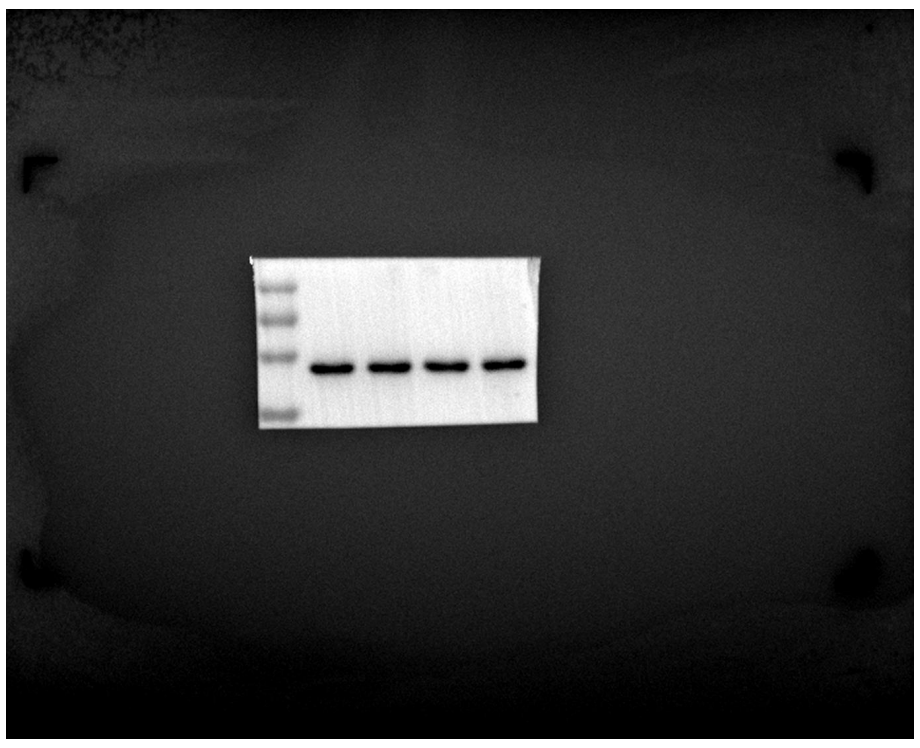

β-actin

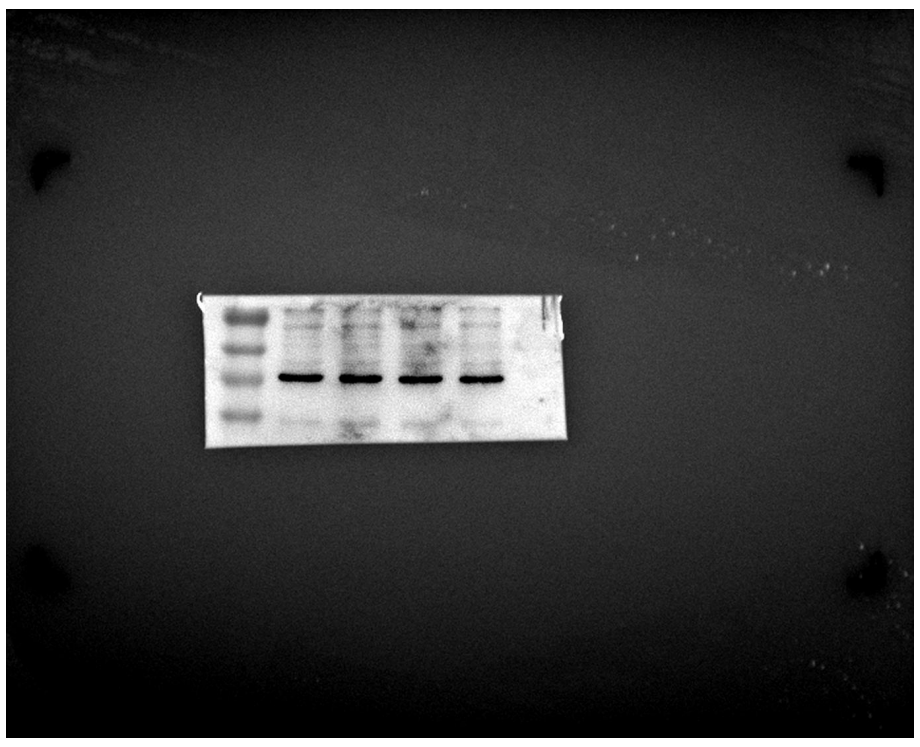

Fig6D

Cyto C

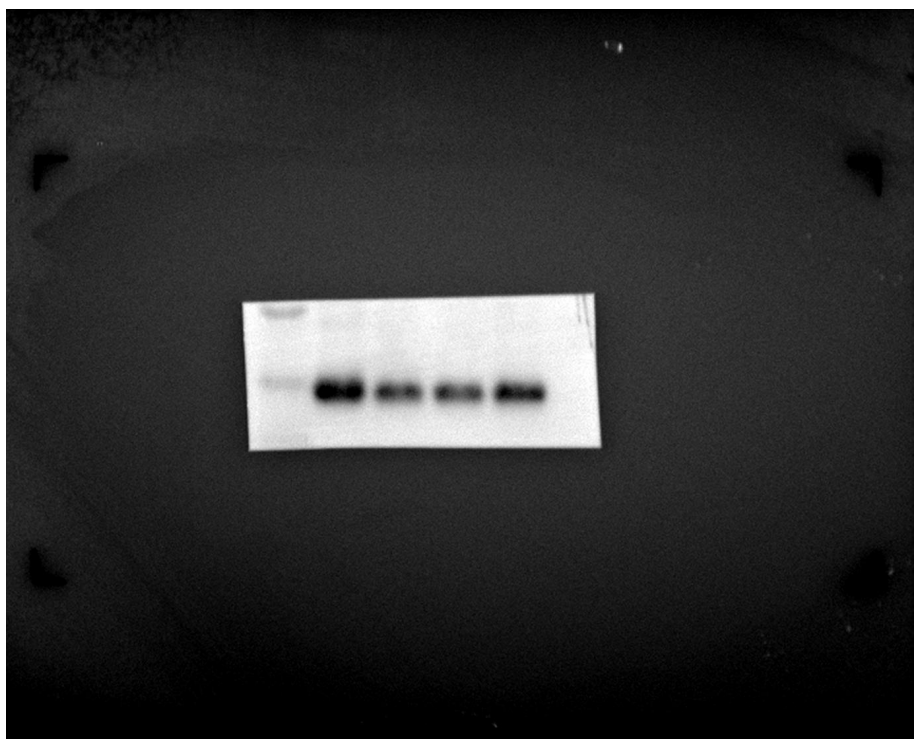

Bax

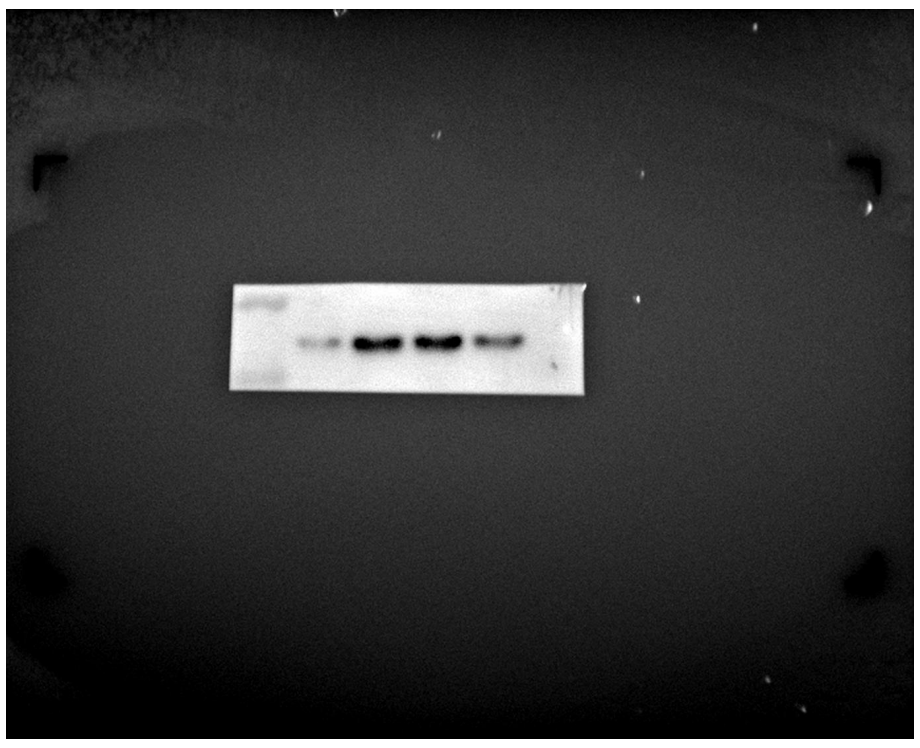

COX IV

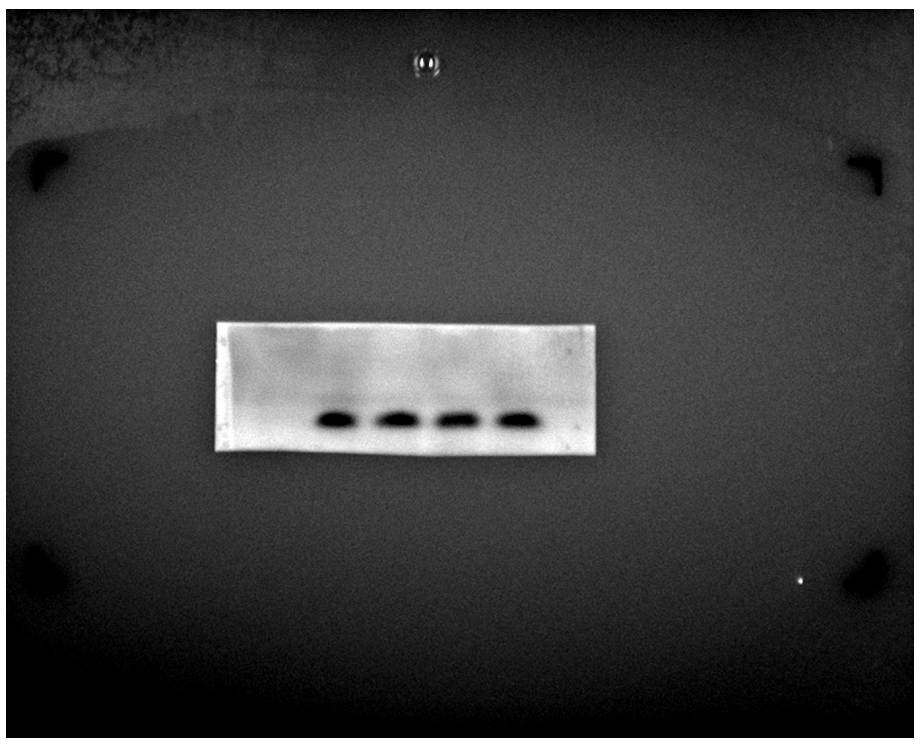

Cyto C

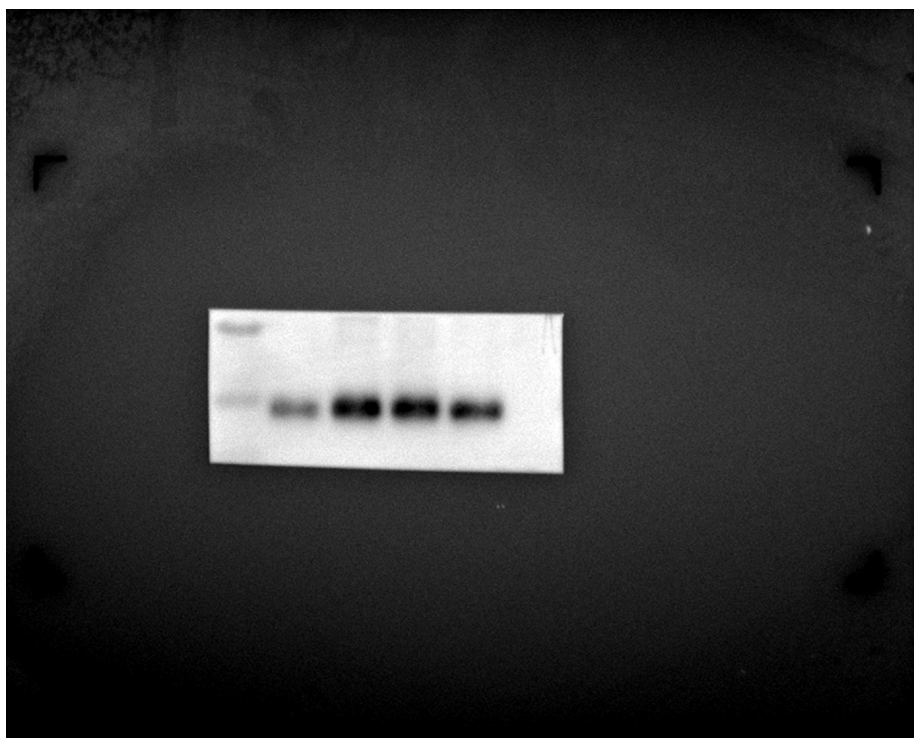

Bax

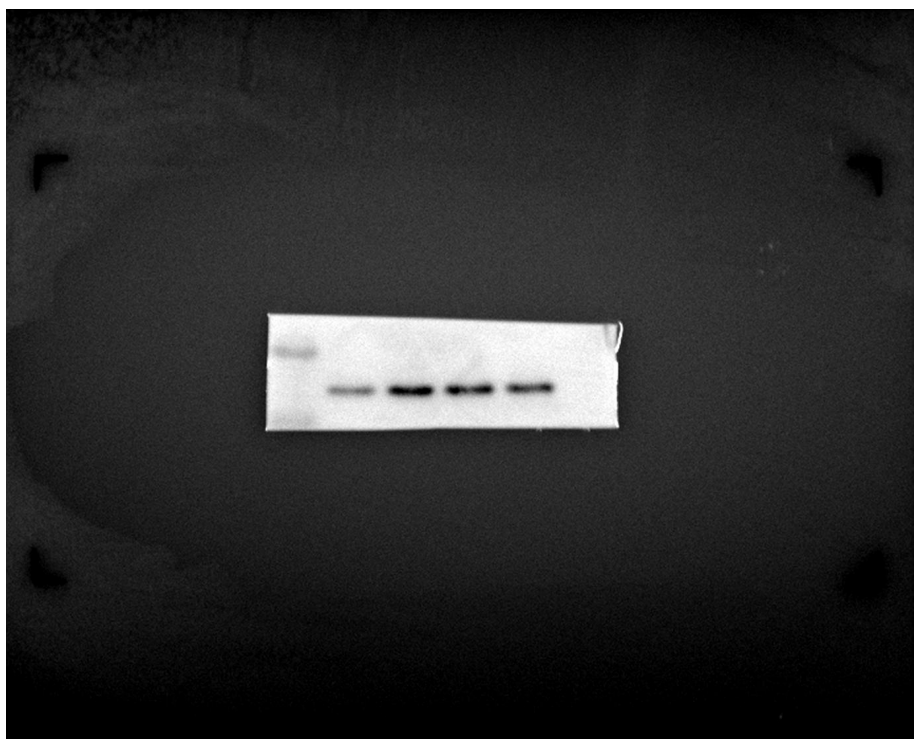

β-actin

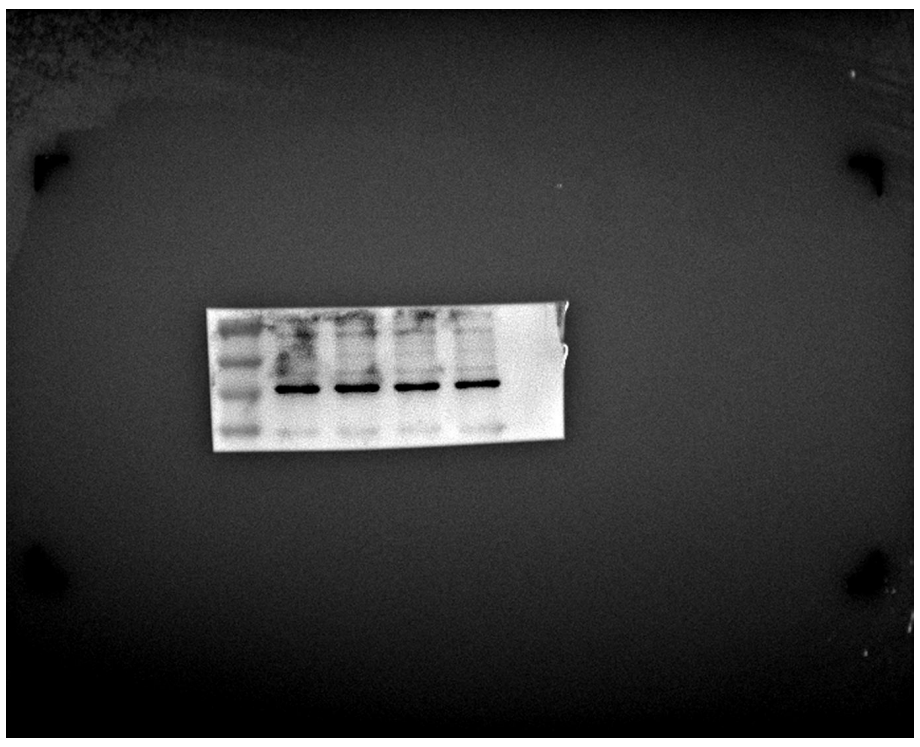

Supplement: Supplementary file 1 [file DataSheet1.pdf]
